# Next-generation lung cancer pathology: Development and validation of diagnostic and prognostic algorithms

**DOI:** 10.1016/j.xcrm.2024.101697

**Published:** 2024-08-22

**Authors:** Carina Kludt, Yuan Wang, Waleed Ahmad, Andrey Bychkov, Junya Fukuoka, Nadine Gaisa, Mark Kühnel, Danny Jonigk, Alexey Pryalukhin, Fabian Mairinger, Franziska Klein, Anne Maria Schultheis, Alexander Seper, Wolfgang Hulla, Johannes Brägelmann, Sebastian Michels, Sebastian Klein, Alexander Quaas, Reinhard Büttner, Yuri Tolkach

**Affiliations:** 1 Institute of Pathology, University Hospital Cologne, 50937 Cologne, Germany; 2 Department of Pathology, Kameda Medical Center, Kamogawa 296-0041, Japan; 3 Department of Pathology Informatics, Nagasaki University, Nagasaki 852-8131, Japan; 4 Institute of Pathology, University Hospital Aachen, 52074 Aachen, Germany; 5 Institute of Pathology, University Hospital Ulm, 89081 Ulm, Germany; 6 German Center for Lung Research, DZL, BREATH, 30625 Hanover, Germany; 7 Institute of Clinical Pathology and Molecular Pathology, Wiener Neustadt State Hospital, 2700 Wiener Neustadt, Austria; 8 Institute of Pathology, University Hospital Essen, 45147 Essen, Germany; 9 Medical Faculty University of Cologne, 50937 Cologne, Germany; 10 Danube Private University, 3500 Krems an der Donau, Austria; 11 University of Cologne, Faculty of Medicine and University Hospital Cologne, Department of Translational Genomics, 50937 Cologne, Germany; 12 Mildred Scheel School of Oncology, Faculty of Medicine and University Hospital Cologne, University of Cologne, 50937 Cologne, Germany; 13 University of Cologne, Faculty of Medicine and University Hospital Cologne, Center for Molecular Medicine Cologne, 50937 Cologne, Germany; 14 University of Cologne, Faculty of Medicine and University Hospital of Colone, Lung Cancer Group Cologne, Department I for Internal Medicine and Center for Integrated Oncology Aachen Bonn Cologne Dusseldorf, 50937 Cologne, Germany

**Keywords:** lung cancer, NSCLC, AI, algorithm, subtyping, prognosis

## Abstract

Non-small cell lung cancer (NSCLC) is one of the most common malignant tumors. In this study, we develop a clinically useful computational pathology platform for NSCLC that can be a foundation for multiple downstream applications and provide immediate value for patient care optimization and individualization. We train the primary multi-class tissue segmentation algorithm on a substantial, high-quality, manually annotated dataset of whole-slide images with lung adenocarcinoma and squamous cell carcinomas. We investigate two downstream applications. NSCLC subtyping algorithm is trained and validated using a large, multi-institutional (*n* = 6), multi-scanner (*n* = 5), international cohort of NSCLC cases (slides/patients 4,097/1,527). Moreover, we develop four AI-derived, fully explainable, quantitative, prognostic parameters (based on tertiary lymphoid structure and necrosis assessment) and validate them for different clinical endpoints. The computational platform enables the high-precision, quantitative analysis of H&E-stained slides. The developed prognostic parameters facilitate robust and independent risk stratification of patients with NSCLC.

## Highlights

• AI-based pipeline allows for explainable analysis of histological slides
• Accurate non-small cell lung cancer subtyping is possible
• Quantitative prognostic parameters provide reliable patient risk stratification
• Developed pipeline can be used in numerous downstream clinical applications

## Graphical abstract

**Figure:**
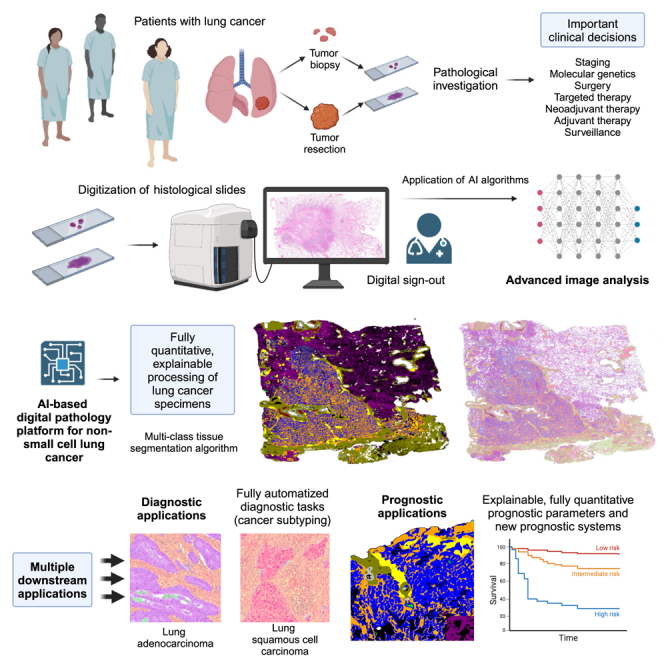

## Teaser

Kludt et al. develop an AI-based digital pathology platform for lung cancer. This advanced platform enables the extraction of explainable features from benign and tumor tissue, facilitating the creation of powerful tools for both improved diagnosis and more accurate assessment of lung cancer aggressiveness and patient prognosis.

## Introduction

**Figure 1:**
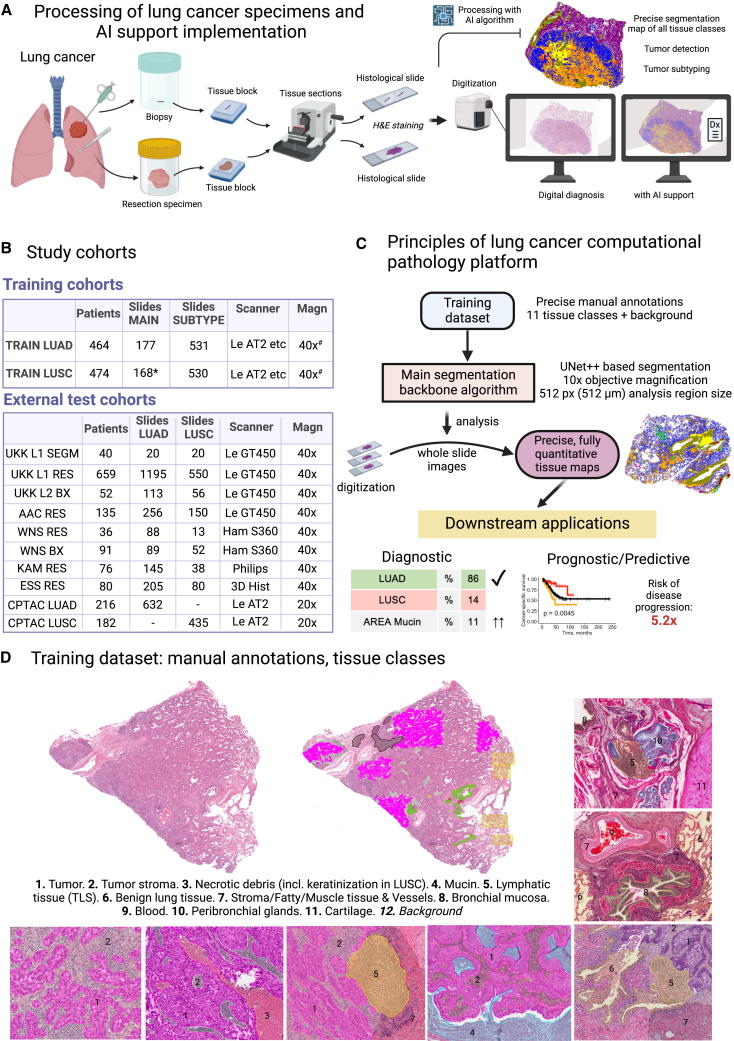
Development of computational platform for non-small cell lung cancer (A) Types of lung cancer specimens and principles of processing in pathology department. Digital pathology allows for diagnostic sign out of cases on the computer monitor and broad application of supportive AI-based tools for automatized slide analysis. (B) Training and external test study cohorts. The slides that were manually annotated and used for development of pixel-wise segmentation algorithms (main algorithm for tissue segmentation and subtyping algorithm) are outlined in “Training cohorts” as MAIN and SUBTYPE, respectively. UKK L1 SEGM is a manually annotated dataset that was used for formal validation of segmentation accuracy. Further datasets were used for validation of non-small cell lung cancer (NSCLC) subtyping algorithm (at slide level). Abbreviations: MAIN, main dataset for multi-class tissue segmentation algorithm; SUBTYPE, NSCLC subtyping algorithm dataset; LUAD, lung adenocarcinoma; LUSC, lung squamous cell carcinoma; Magn, magnification; Scanner, Le – Leica Aperio; Ham, Hamamatsu Nanozoomer; 3D Hist, 3D Histech; UKK, University Hospital Cologne; AAC, University Hospital Aachen; WNS, Hospital Wiener Neustadt; KAM, Kameda Medical Hospital; ESS, University Hospital Essen; CPTAC, Clinical Proteomic Tumor Analysis Consortium cohort. ∗ 107 slide were fully annotated as shown in (D), 61 slide were additionally annotated for underrepresented tissue classes; ^#^ most of the slides were 40x; some of the slides were scanned under 20x magnification. (C) The main segmentation algorithm was developed using a high-quality large manually annotated dataset. This can be used for quick, primary processing of the whole-slide images and creation of precise, fully quantitative tissue maps. These allow a multiple number of downstream applications, diagnostic (e.g., subtyping) or prognostic/predictive. (D) Principles of high-precision, extensive manual annotations with representative examples of 11 tissue classes are presented. The figure was prepared with BioRender. See also Table S1.

Non-small cell lung cancer (NSCLC) is the second most frequent and the deadliest epithelial cancer.^1^ NSCLC accounts for more than 80% of all lung cancers with two main histological subtypes: lung adenocarcinoma (LUAD) and lung squamous cell carcinoma (LUSC).^2^ A higher incidence of LUAD is reported in female patients (57% vs. 39% in males, among all lung cancer subtypes) with an opposite trend for LUSC (25% in males vs. 12% in females).^2^ Two main types of specimens are routinely processed in pathology departments: biopsies and resection specimens (Figure 1A). Although the basic principles of tissue processing up to the preparation of hematoxylin and eosin (H&E)-stained tissue sections remain the same for many decades, a profound transformation of the diagnostic process can be observed in modern pathology departments. The introduction of digital pathology allows not only to diagnose the cases on a computer monitor without a microscope but also application of the image analysis tools that can substantially automatize, optimize, and improve the diagnostic process (Figure 1A).^3^

AI-based approaches to image analysis might be a foundation for useful diagnostic, prognostic, and predictive tools in pathology and oncology.^3^^,^^4^^,^^5^^,^^6^^,^^7^ Although the feasibility of developing these tools to equal accuracy as human pathologists was shown for almost every malignant tumor, only a few of them are extensively clinically validated^8^^,^^9^^,^^10^^,^^11^^,^^12^^,^^13^^,^^14^^,^^15^ or available in the market.^4^ More than 99% of research studies published on this topic do not reach any clinical development at all. The reasons for this are manifold: (1) the necessity of large, very high-quality training datasets, which require a lot of effort to create, (2) poor generalization of tools to unseen data, (3) absence of adequate clinical validation, (4) the problem of quality control still insufficiently addressed,^16^ and (5) explainability in digital pathology, among others.

Several recent developments are being extensively evaluated with a hope to boost the precision of AI algorithms for digital pathology, such as foundation models^17^^,^^18^^,^^19^^,^^20^ which act as powerful feature extractors from pathology images but also weakly supervised approaches in areas where annotations are limited^21^^,^^22^^,^^23^ and integrative/generalistic (including agentic and vision language) approaches.^17^^,^^24^^,^^25^ Still, in lung cancer compared to other tumors, e.g., prostate cancer, almost all studies concerning tumor detection approached this in binary mode (tumor vs. non-tumor tissue) or with classification models.^26^^,^^27^^,^^28^^,^^29^^,^^30^^,^^31^ Classification algorithms analyze slides in tiles (regions), which are obsolete as diagnostic tools due to low analysis resolution. To date, no H&E-based tools are available for lung cancer. This, in turn, hampers further development of prognostic and predictive tools to optimize and personalize patient care.

In this study, based on the largest, high-quality training dataset available to date (Figure 1B), we developed a potent computational pathology platform for NSCLC (Figure 1C) that can quickly analyze H&E-stained whole-slide images (WSIs) of resection and biopsy specimens and precisely segment all relevant tumor and benign tissue classes (*n* = 11) at a pixel level. This platform can be used for many diagnostic, prognostic, and predictive downstream applications. We show that the platform can be immediately leveraged concerning its clinical value in two downstream applications. Firstly, we train an accurate diagnostic model for NSCLC subtyping and validate it using a large external, independent, multi-institutional cohort of patient cases involving expert pathologists in comparison. Secondly, we suggest four fully quantitative, explainable, independent, capable prognostic parameters derived from H&E-stained tissue samples that allow prognostic stratification of patients with resectable NSCLC concerning tumor progression and survival. We release publicly three datasets to facilitate international efforts in algorithm development and interoperability in the domain of lung cancer.

## Results

### Development of NSCLC computational pathology platform

The key part of the developed lung cancer platform is a potent multi-class (*n* = 11) tissue segmentation algorithm. This was developed using a high-quality, manually annotated, heterogeneous, multi-institutional dataset including 345 WSIs with LUAD and LUSC cases (datasets: Figure 1B; platform principle and algorithms: Figure 1C; annotation principle: Figure 1D); for details, see STAR Methods.

#### Formal validation of the model segmentation accuracy

**Figure 2:**
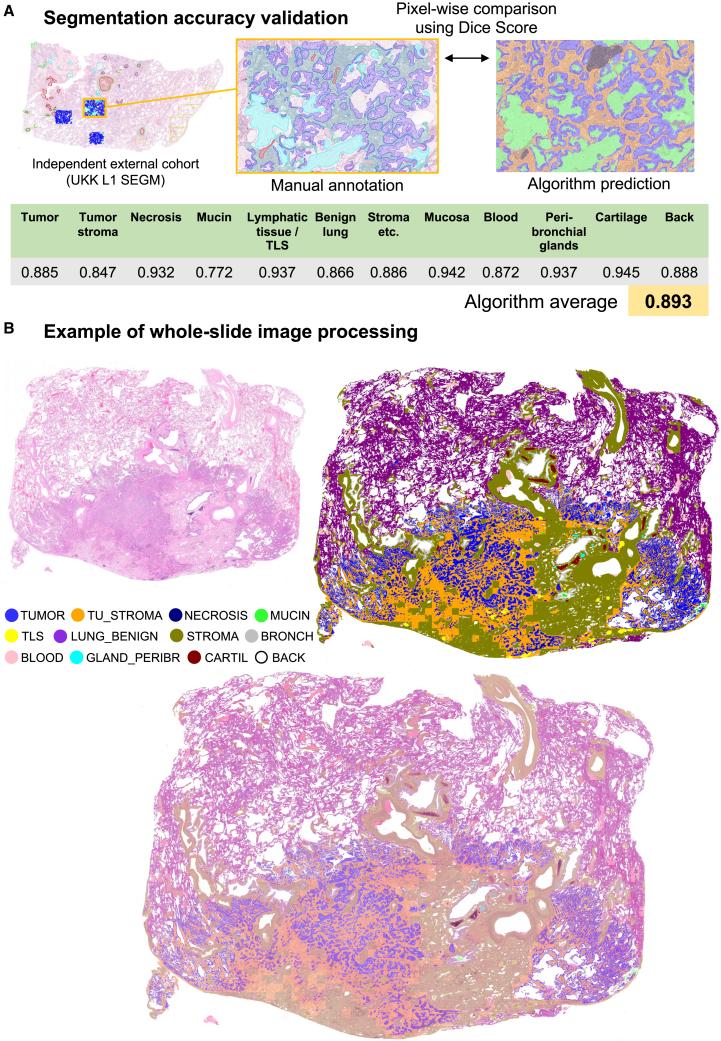
Validation of multi-class tissue segmentation accuracy and example of WSI processing by the backbone algorithm of platform (A) Segmentation accuracy was tested using an external cohort. Forty whole-slide images (patients *n* = 40) representative of the broad spectrum of morphologies and histological grades were extensively manually annotated (LUAD *n* = 20, LUSC *n* = 20). Dice metrics (intersection of annotated and predicted regions, with score = 1.0 representing ideal quality of segmentation) were calculated for each tissue class representing excellent quality of segmentation. Most inaccuracies were related not to the obvious false positive/false negative results but to a pixel-level variations of the “perceived” borders of the different tissue structures that are not relevant. (B) Example of processing of whole-slide image of lung adenocarcinoma (KAM RES cohort) showing original image, segmentation mask produced by algorithm, and overlay of the mask on the original image. Fully quantitative and precise “decoding” of image is possible with algorithm that can be used for further downstream applications. Class abbreviations: TUMOR, epithelial tumor component; TU_STROMA, tumor stroma; NECROSIS, necrotic debris; MUCIN, mucin; TLS, lymphatic tissue/tertiary lymphoid structures; LUNG_BENIGN, tumor-free lung parenchyma; STROMA, non-tumor-associated stroma, fat, vessels, and muscle tissue; BRONCH, bronchial mucosa; BLOOD, areas of bleeding or erythrocytes; GLAND_PERIBR, peribronchial mucous glands; CARTIL, cartilage; BACK, slide background.

Forty WSIs (University Hospital Cologne/UKK L1 SEGM independent dataset, Figure 1B) representative of different NSCLC subtypes/morphologies were extensively annotated (see STAR Methods). The annotated regions were analyzed by the trained model to test segmentation accuracy. The metrics show excellent segmentation quality of all classes (overall average Dice score = 0.89; Figure 2A). Most inaccuracies are related not to the obvious false positive/false negative results but to pixel-level variations of the “perceived” borders of the different tissue structures.

### Computational pathology platform for NSCLC

**Figure 3:**
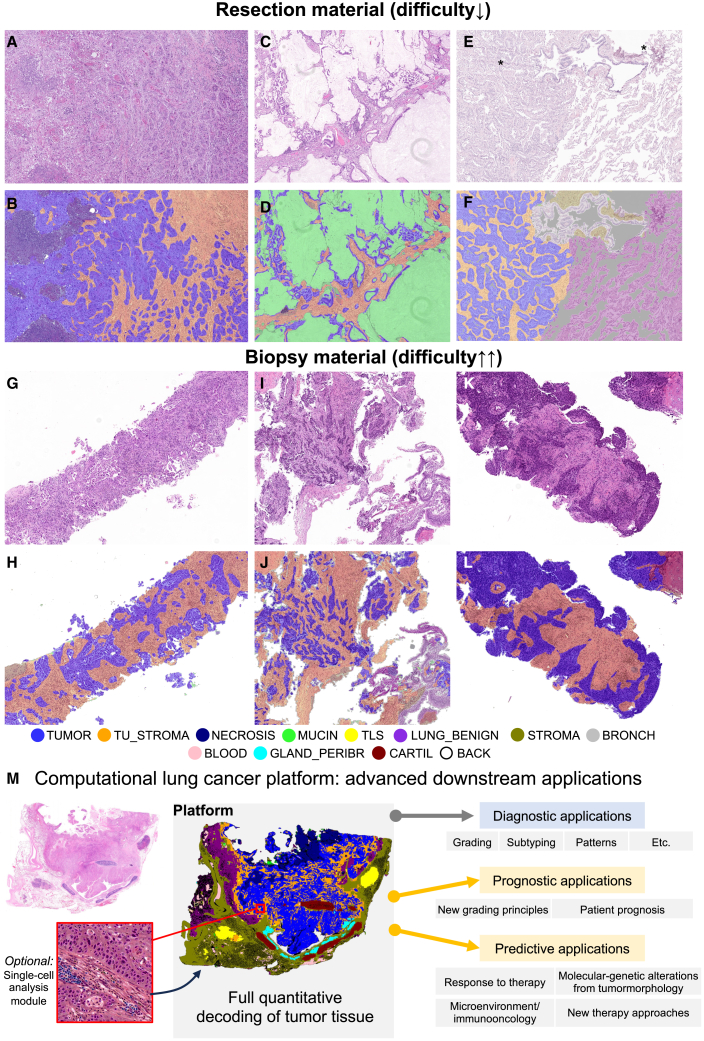
Examples of platform application to resection and biopsy cases (A–F) Resection cases (A, C, and E) and corresponding overlays with multi-class tissue mask (B, D, and F). Cases (A) and (C) are from Wiener Neustadt (WNS) RES cohort; (E) UKK L1 RES cohort (note artificial changes∗ that are processed correctly by the algorithm). Resection cases are less challenging as they normally have much fewer tissue-processing artifacts (mechanical artifacts). (G–L) Biopsy cases (G, I, and K) and corresponding overlays produced by algorithm (H, J, and L). All cases are from WNS BX cohort. Naturally, biopsy samples are a very difficult material due to high levels of artificial changes; however, the segmentation quality for tumor tissue was estimated as excellent by participating pathologists, allowing usage of the platform on biopsy material as well. Uniformly excellent quality independent of cohort was evident in resection specimen cohorts, as estimated visually by participating expert pathologists. (M) Developed computational platform, optionally connected to single-cell detection/classification algorithms (available open-source), can be used for a large number of downstream applications. Examples of potential downstream applications are provided.

The developed platform allows full quantitative analyses of NSCLC WSIs. The example of WSI processing is presented in Figure 2B. The segmentation model quickly provides a high-precision segmentation mask of an image, especially that of the tumor region that includes four main tissue classes (epithelial tumor component, tumoral stroma, necrotic debris, and mucin). All the detected classes can be quantitatively and spatially analyzed. A large multi-institutional cohort of NSCLC cases from 5 different pathology institutes from three countries (Figure 1B) and one open-source (Clinical Proteomic Tumor Analysis Consortium [CPTAC]) cohort were gathered for processing with a model (including two biopsy cohorts). The examples of processing with segmentation model of both resection and biopsy cases are provided in Figure 3. The computational pathology platform can be used for many downstream applications (Figure 3M). We study in detail one diagnostic application (NSCLC subtyping) and one prognostic application showing immediate benefit for pathologists and the possibility of developing new, potent, fully quantitative prognostic parameters from analyzed images.

### Development of NSCLC subtyping algorithm

**Figure 4:**
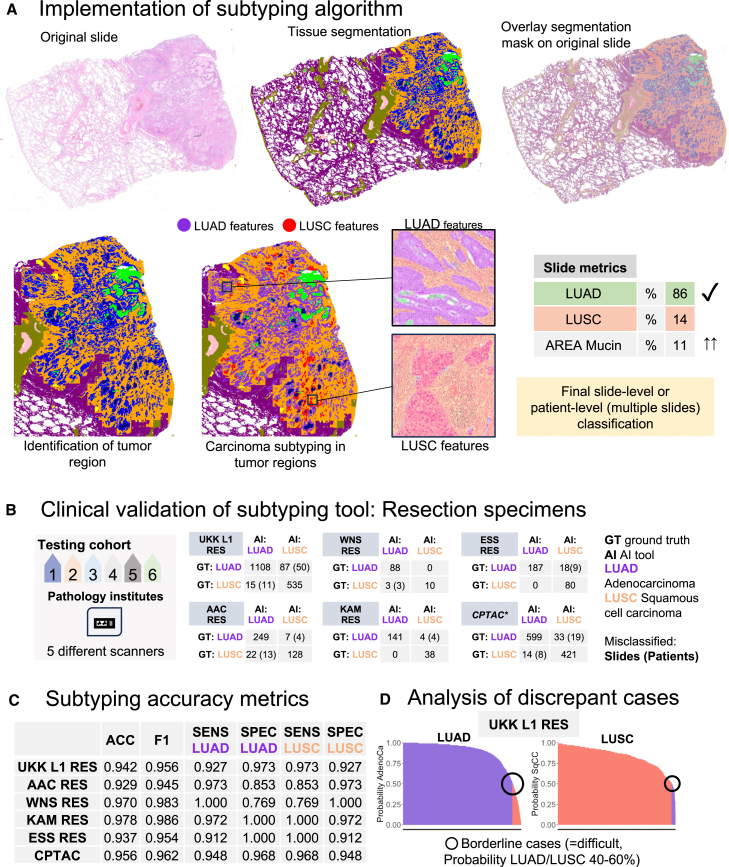
Development and evaluation of diagnostic downstream algorithm for NSCLC subtyping (A) The modus operandi of the subtyping algorithm includes analysis of tumor regions and epithelial tumor component detected by a platform’s backbone multi-class tissue segmentation algorithm. Precise mapping of glandular and squamous features is available for explainability to pathologists. Also precise quantifications of features are provided with resulting slide-level classification of tumor as adenocarcinoma or squamous cell carcinoma. Mucin is detected in form of intraglandular component or mucin lakes with quantification of mucin area within tumor that allows easy identification of mucinous and colloid adenocarcinoma subtype by pathologists. In case of multiple slides, metrics are provided per slide and per case. (B) Clinical validation of subtyping algorithm using large multi-institutional cohort of resection cases digitized by five most common scanning systems (institutes *n* = 6, patients *n* = 1,384, slides *n* = 3,787 including LUAD/LUSC 2,521/1,266). Slide-level classification is provided in confusion tables (with case level for misclassified slides). (C) Subtyping accuracy metrics at slide level for single cohorts. Abbreviations: ACC, overall accuracy; F1, F1 score; SENS, sensitivity; SPEC, specificity; Ad, Adenocarcinoma; SqCC, squamous cell carcinoma. (D) Analysis of per-slide area probability distributions (% area classified as LUAD or LUSC; the largest UKK L1 RES cohort of patients). Only single slides show borderline probabilities around 50% (40%–60%) while most cases are classified as a subtype with high levels of certainty. Most cases with borderline probabilities represent challenging cases (poorly differentiated solid carcinomas) which are not solvable without additional immunohistochemistry studies. Such cases are additionally investigated (Figures 5D–5F) with inclusion of five expert pathologists. See also Figures S1–S12.

All The Cancer Genome Atlas (TCGA) lung cancer cases were used for training of the subtyping model, leveraging the developed auto-annotation pipeline based on the main algorithm (see STAR Methods). The training was carried out similarly to the main segmentation algorithm with a reduced number of only tumor-related classes (LUAD, LUSC, necrosis, tumor stroma, mucin). The principle of work for the NSCLC subtyping algorithm is outlined in Figure 4A.

### External validation of NSCLC subtyping algorithm: Resection cases

Six external independent cohorts of resection cases with a total of 3,787 images (LUAD *n* = 2521, LUSC *n* = 1266) were available for model validation (Figures 1B and 4B). The model showed high overall accuracy for subtyping lung cancers into LUAD and LUSC (overall accuracy 0.929–0.978, F1 score 0.927–0.886 over all six cohorts) with high levels of sensitivity and specificity (Figure 4C). Detailed analysis of discrepant slides/patient cases is provided in Figures 4B and 4D. Visual analysis of discrepant cases showed that virtually all misclassifications were related to poorly differentiated solid tumors which are naturally challenging cases for a decision based on H&E stains only. Importantly, when final classification probabilities of single slides derived from the subtyping model were analyzed in the largest, most representative cohort (UKK L1 RES; slides *n* = 1745), only a few cases were in the “borderline” subtype probabilities (for both LUAD and LUSC probability 40%–60%) with most cases having high probabilities for a proper subtype indicating high levels of algorithm certainty. Model-derived probabilities and maps (Figure 4A) are useful for the explainability of decisions and can support pathologists during the diagnostic process.

### External validation of NSCLC subtyping algorithm: Biopsy cases

**Figure 5:**
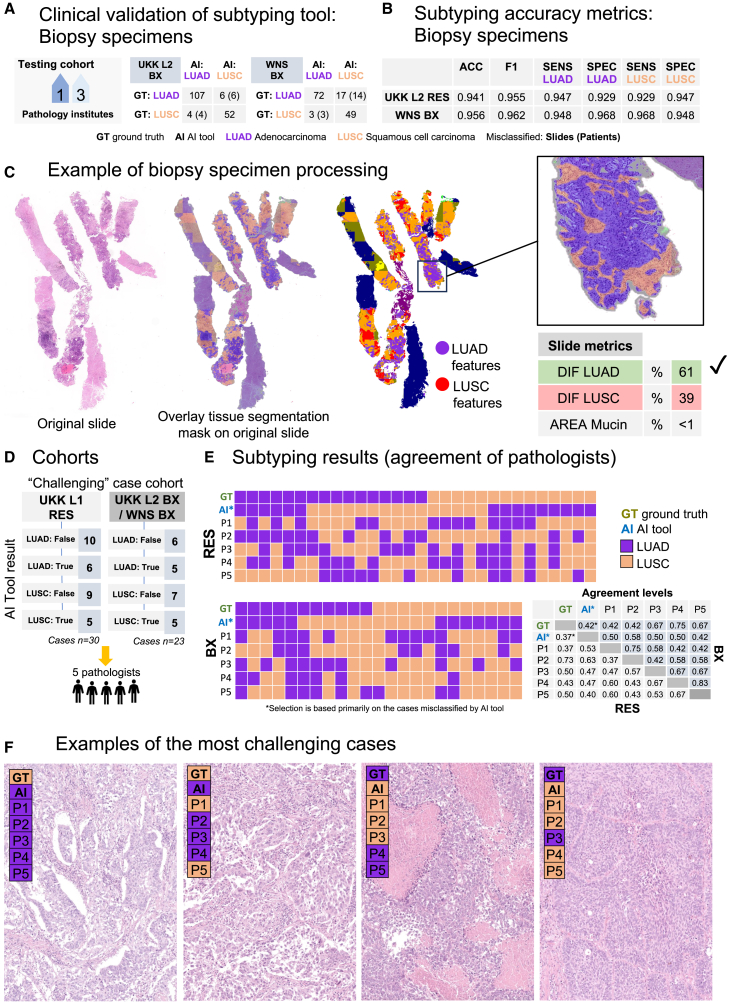
Clinical validation of subtyping algorithm in biopsy cases and detailed investigation of “challenging” cases (A) Clinical validation of subtyping algorithm using two cohorts of biopsy cases digitized by two different scanning systems (slide *n* = 310 [LUAD/LUSC 202/108], patients *n* = 143). Slide-level classification is provided in confusion tables (with case level for misclassified slides). (B) Subtyping accuracy metrics at slide level for single cohorts. Abbreviations: ACC, overall accuracy; F1 – F1 score; SENS, sensitivity; SPEC, specificity; Ad, adenocarcinoma; SqCC, squamous cell carcinoma. (C) Example of algorithm processing of the biopsy case with visual outputs and quantitative metrics for classification of the case. Same color coding as in Figure 2B. (D) Two cohorts of “challenging” cases (cases misclassified by algorithm and borderline cases), resection and biopsy, were created. These cases were evaluated by five expert pathologists (P1–P5). (E) The results of subtyping by pathologists separately for resection (RES) and biopsy cohorts (BX) are shown with ground-truth classification result from pathology report (including immunohistochemistry evaluation) and AI tool prediction. Note that the cases were intentionally selected that were misclassified by the AI tool. High levels of deviation from the ground truth and interobserver variability were evident for all pathologists (P1–P5) implying that these cases could be only resolved reliable with a help of immunohistochemistry. Confusion table with simple agreement levels is provided on the right side, separately for RES and BX cohorts. (F) Examples of the most challenging cases with subtyping results provided by pathologists, AI tool, and ground truth (GT, immunohistochemistry).

Two external independent biopsy cohorts were available with a total of 310 images (LUAD *n* = 202, LUSC *n* = 108) (Figures 1B, 5A, and 5B). Slightly lower, however, still high levels of classification accuracy were received with overall accuracy and F1 scores of 0.941 and 0.956 and 0.955 and 0.062 for UKK L2 BX and Wiener Neustadt (WNS) BX cohorts, respectively. Details on specificity and sensitivity as well as per-slide classification results are provided in Figures 5A and 5B. An example of subclassification processing by the algorithm in a biopsy case is outlined in Figure 5C.

### Comparison of algorithm performance with foundation encoder-based models and weakly supervised method

Weakly supervised approaches for such tasks as tumor subtyping are gaining popularity. The reason for this is the possibility of training a model using only slide-level labels, not involving any manual annotations from pathology experts. Moreover, several pathology data-pretrained foundation models were released recently that are potent feature extractors from images and are supposed to facilitate development of more accurate diagnostic algorithms. Firstly, we train a simple model using one of two state-of-the-art foundational feature extractors (UNI and Prov-GigaPath) and additional simple dense and classification layers (STAR Methods). We show that our fully supervised pixel-wise segmentation model provides better subtyping accuracy on patch level (AUROC 0.946 vs. 0.923 for UNI and 0.901 for Prov-GigaPath for full training data; see Figure S1 for details, including training on limited amount of data). Next, we select UNI trained on a full dataset and show the superiority of our supervised method on the slide level in most of the test datasets (Figure S2). Significant accuracy drops can be seen for the UNI-based model in biopsy datasets (AUROC 0.985 vs. 0.883 for own vs. UNI model on UKK BX and 0.944 vs. 0.844 for WNS BX, respectively; details in Figure S2). Next, we train a model based on the CLAM principle and using the UNI feature extractor (STAR Methods). Importantly, the CLAM/UNI-based model reaches AUC levels comparable to our segmentation-based method in some but not all resection datasets but still significantly underperforms in the biopsy setting (Figure S5).

Detailed morphological analysis of simple classifiers and CLAM-based methods was performed. For the former, restricted contextual information in single patches due to the small patch sizes of foundation models might be among most important problems (Figures S3 and S4). For the latter, analysis revealed that a substantial number of high attention scores are from completely benign tissue (e.g., necrosis, cartilage, benign mucosa, metaplasia, etc.), which are less relevant and influential for the final classification in the resection setting (given high tumor volume); however, they are unacceptable and distract slide-level classifications for the biopsy setting (Figures S6–S11). These obviously represent statistical/correlative biases and are a significant limitation of the CLAM learning principle. The clearest CLAM classifications for LUAD were in areas with glandular differentiation while misclassifications as LUSC were (like supervised models) solid areas (Figure S12), indicating no additional value from CLAM in this context.

### Analysis of challenging cases (agreement with expert pathologists)

To further analyze the performance of the algorithm compared to human pathologists, we created two cohorts of challenging cases (intentionally approximately 2/3 of the cases are the cases where the AI algorithm misclassified as well as cases with borderline classification), one for resection specimens and one for biopsy specimens (Figure 5D). Five expert pathologists regularly working with lung cancer cases were asked to provide the subtype for all cases of the two cohorts (H&E-stained slides only, Figure 5E). The results show low levels of classification accuracy (resections agreement for five pathologists 0.37–0.73, biopsy 0.42–0.75; ground truth revealed by immunohistochemistry) and very high levels of interobserver variability (Figure 5E), one more time emphasizing that such cases are not resolvable without additional immunohistochemistry. The examples of the most challenging cases are shown in Figure 5F.

### Prognostic image biomarkers with independent prognostic value

**Figure 6:**
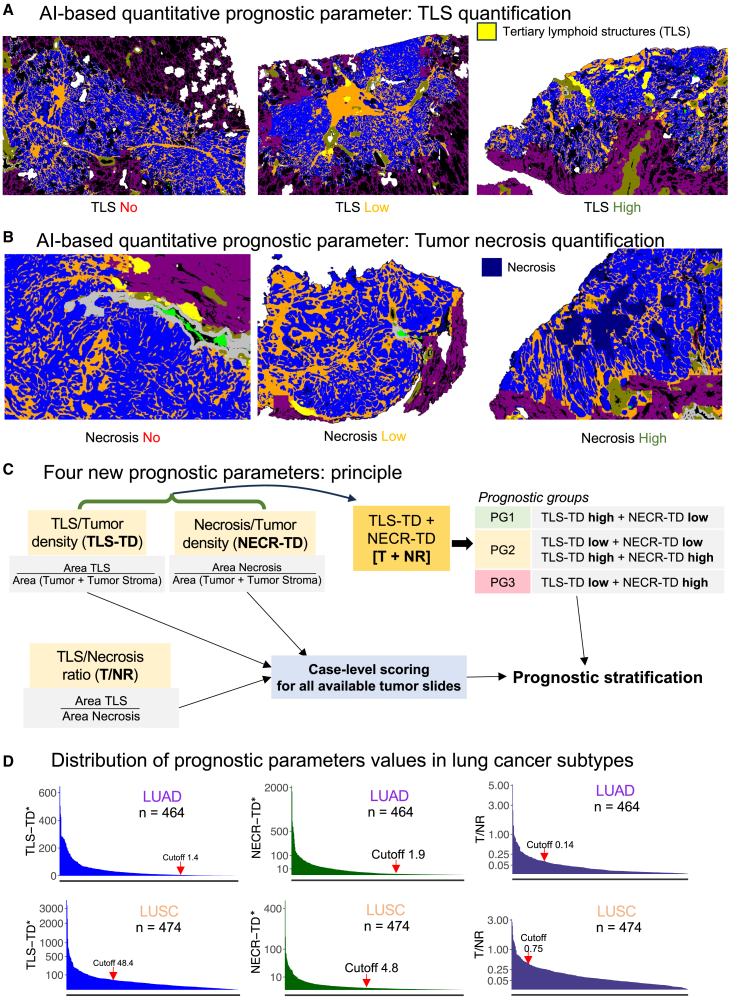
Development of AI-based, quantitative prognostic parameters (A) AI-based quantification of tertiary lymphoid structure (TLS) density. Three lung cancer cases are shown with yellow regions corresponding to detected TLS objects. Other colors as in Figure 2B. (B) AI-based quantification of intratumoral necrosis density. Three lung cancer cases are shown with navy blue regions corresponding to detected necrosis regions. Other colors as in Figure 2B. (C) Principle of quantification for four new prognostic parameters: TLS tumor density (TLS-TD), necrosis tumor density (NECR-TD), TLS/necrosis ratio (T/NR), and cumulative score with three prognostic group based on combination of TLS-TD and NECR-TD. T/NR and cumulative scores are compound parameters based on TLS and NECR quantification in the tumor. The parameters are fully explainable and do not involve any “black box” features from deep learning algorithm. The parameters TLS-TD, NECR-TD, and T/NR are dichotomized using identified optimal cutoff to derive prognostic subgroups. (D) Distribution of TLS-TD, NECR-TD, and T/NR values among LUAD and LUSC cases of the prognostic test cohort. In each plot each measurement is a case-level value of the corresponding parameter. ∗ TLS-TD for LUAD/LUSC and NECR-TD for LUSC are linearly upscaled using x1,000 multiplication (NECR-TD for LUSC using x100) for easiness of perception. Red arrows represent identified best risk stratification cutoffs for corresponding parameters used later for prognostic analyses (Figure 7). See also Tables S2–S7.

The developed computational platform for NSLSC allows for highly precise, quantitative, and objective analysis of tumor morphology. We show how immediate value concerning the prognosis of patients with LUAD and LUSC can be extracted in a fully explainable and quantitative way from H&E-stained WSIs of patients’ tumors. Tumor necrosis mirrors the biological aggressiveness of the tumor with large areas of necrosis indicating quickly growing and aggressive tumors (Figure 6B). On the other hand, tertiary lymphoid structures (TLSs) are a limiting factor for tumor growth; however, they are still understudied in malignant tumors (Figure 6A). We introduce four new prognostic parameters (Figure 6C) that can be quantitatively derived by the developed platform from H&E images: density of necrosis for whole tumor area (NECR-TD), density of TLS for whole tumor area (TLS-TD), a direct ratio between TLS and necrosis areas (T/NR), and cumulative prognostic group classifier (T + NR) based on NECR-TD and TLS-TD values (three prognostic groups; Figure 6C). The distribution of the TLS-TD, NECR-TD, and T/NR parameters in patients with LUAD and LUSC is shown in Figure 6D.

**Figure 7:**
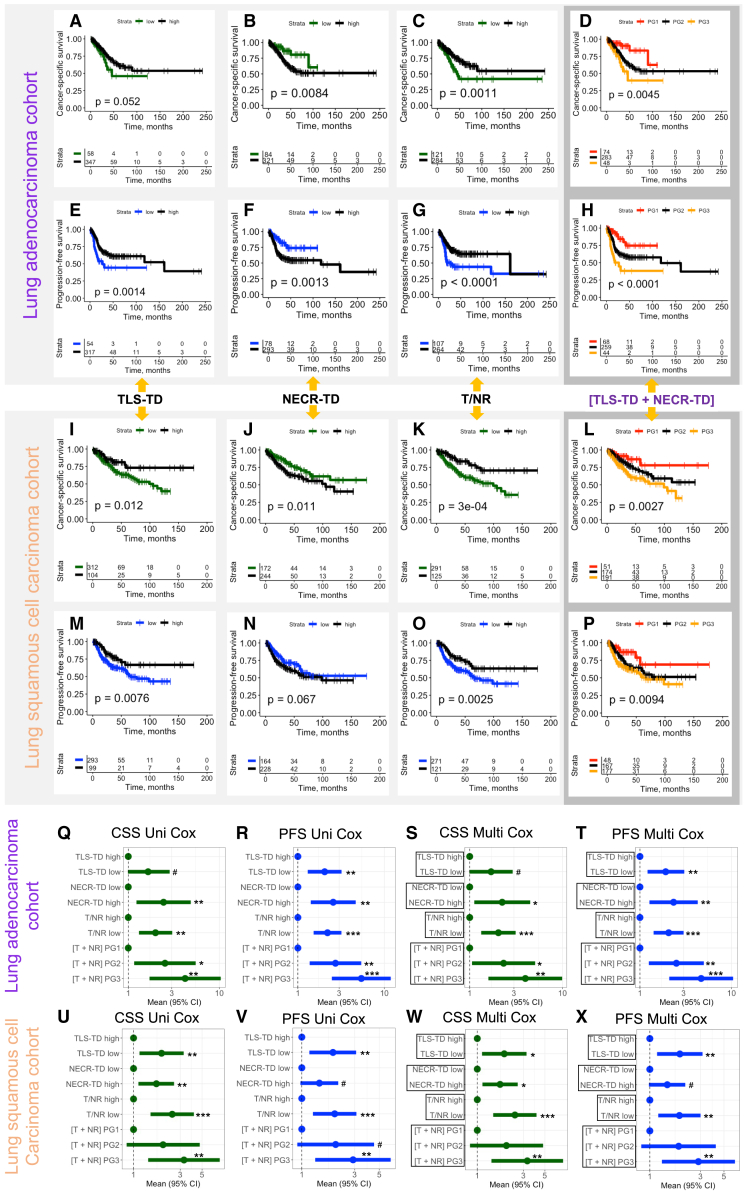
Evaluation of prognostic role of AI-based prognostic parameters for cancer-specific and progression-free survival endpoints (A–H) Lung adenocarcinoma cohort: (A and E) TLS-TD, (B and F) NECR-TD, (C and G) T/NR, (D and H) [T + NR]. (I–P) Lung squamous cell carcinoma cohort: (I and M) TLS-TD, (J and N) NECR-TD, (K and O) T/NR, (L and P) [T + NR]. The parameters TLS-TD, NECR-TD, and T/NR are dichotomized using identified optimal cutoff to derive prognostic subgroups (same cutoff for all prognostic endpoints). (Q–X) Results of univariate and multivariate Cox proportional hazard model analysis for new prognostic parameters concerning cancer-specific survival (CSS) and progression-free survival (PFS). All parameters show independent prognostic value in multivariate analysis including common prognostic variables. (Q–T) Lung adenocarcinoma cohort. (U–X) Lung squamous cell carcinoma cohort. Comment: all multivariate models always include pT and pN classification of the tumor and one prognostic parameter; therefore, one plot shows several multivariate models, one for each of prognostic parameter for easiness of visualization. The analyzed parameter is included in frame. Plots show hazard ratios (HRs) and 95% confidence interval (95% CI). #*p* value 0.05–0.1 (statistical trend), ∗*p* value 0.01–0.05, ∗∗*p* value 0.001–0.01, ∗∗∗*p* value < 0.001. Detailed information to univariate and multivariate Cox analysis is provided in Tables S2–S7. See also Figure S13.

Importantly all four parameters show independent prognostic value (when analyzed together with common pathological variables pT and pN) for cancer-specific and progression-free survival (PFS), for both LUAD (Figures 7A–7H) and LUSC (Figures 7I–7P). Interestingly, the modus operandi of prognostic stratification is slightly different in LUAD and LUSC. As for TLS-TD, it allows for the identification of a smaller subset of patients with unfavorable outcomes in LUAD (TLS-TD low) and with favorable outcomes in LUSC (TLS-TD high) (Figures 7A–7E, 7I, and 7M). Notably, the combination of the two density parameters (TLS-TD, NECR-TD) either as a direct ratio (T/NR) or as a stratification measure (T + NR; PG 1–3) allows for even better stratification of patients with good, moderate, and poor prognosis, which is independently valuable for prognosis estimation with cancer-specific survival (CSS) and PFS as endpoints in multivariate Cox analysis (Figures 7C, 7D, 7G, 7H, 7K, 7L, 7O, and 7P). For overall survival, all parameters show independent prognostic value in the LUAD cohort, with only the T/NR ratio being independently prognostically relevant in the LUSC cohort (Figure S13). Detailed outputs of univariate and multivariate Cox analysis are provided in Tables S2–S7.

## Discussion

Digital pathology and computational analysis of histological images revolutionize the field of diagnostic pathology. Still, there are only a few potent instruments that allow for such analysis. In this study, we develop a potent computational pathology platform (Figure 1C). The backbone of the platform is a highly accurate multi-class tissue segmentation algorithm (Figure 1D). The model was developed using a large, heterogeneous, multi-institutional, high-quality manually annotated dataset (Figures 1B and 1D). Compared to previous deep learning algorithms for processing WSIs,^32^ our algorithm represents a new quality/generation of lung cancer approach concerning precision and accuracy. Importantly, only a limited number of studies have been published to date regarding tissue detection/classification in WSIs from lung cancer patients.^26^^,^^27^^,^^28^^,^^29^^,^^30^ Most of them^26^^,^^28^^,^^29^ excluding one small study^27^ utilize classification neural networks analyzing the WSIs in coarse regions, which are obsolete for diagnostic application due to a very low analysis resolution. Our algorithm builds highly precise segmentation maps. Moreover, most of the published studies^26^^,^^28^^,^^29^^,^^30^ utilize a binary principle of detecting tumor vs. benign tissue which is not sufficient. Our algorithm can identify all relevant classes (*n* = 11) allowing any form of quantitative analysis (Figures 1D, 2, and 3). In one computational challenge,^30^ the best algorithm trained on the small dataset for binary segmentation (tumor vs. benign) achieved a Dice score for tumor class of 0.8372 (internal validation subset with coarse annotations of tumor regions). Our algorithm achieves a substantially higher Dice score for epithelial-only tumor segmentation (0.885) using a fully independent test dataset (Figure 2A)—by a significantly more difficult task. It performs well in resection and biopsy specimens (Figures 3A–3K).

The developed platform can be considered a potent tool for the development of downstream diagnostic, prognostic, and predictive algorithms (Figure 3M). As an example of a diagnostic downstream algorithm, we develop an NSCLC subtyping tool (LUAD/LUSC and detection of mucinous component) (Figures 4 and 5). The algorithm shows glandular and squamous tumor “features” to pathologists in an explainable way (Figures 4A and 5C). We show high levels of H&E-only subtyping accuracy using 6 independent, external cohorts of resection and biopsy cases (Figures 4B, 4C, 5A, and 5B). A broad range of accuracies for distinguishing between LUAD and LUSC was reported in previously published studies from 75% to >99%, mostly for non-independent or small test datasets^23^^,^^33^^,^^34^^,^^35^; for systematic review see also Davri et al.^32^ In our study, the largest dataset (of six) includes 1,745 slides from all consecutive resection cases of one large lung cancer referral center (UKK L1 RES) for five years, representing all possible stages, grades, and morphologies. Moreover, in an additional experiment including 5 experienced pathologists, we show that the nature of misclassified cases precludes their proper classification without immunohistochemistry (Figures 5D and 5E).

Importantly, we perform additional experiments to directly compare our fully supervised approach to two additional approaches involving state-of-the-art foundational extractors (UNI^18^ and Prov-GigaPath^19^) and one state-of-the-art weakly supervised approach (CLAM^23^). We show that the latter approaches can provide very competitive results, especially CLAM-based approach with UNI extractor (Figures S1 and S2). CLAM/UNI models achieved accuracy comparable to our model in some of the resection datasets, including our most representative dataset (UKK RES); however, our analysis revealed several limitations of the model implying the significant superiority of the fully supervised approach. Thus, CLAM-based models learned many statistical biases from the slides leading to high-attention areas in benign regions of almost every slide. While being “compensated” in resection slides due to large volume of tumor tissue, they are obviously distracting and result in numerous false classifications in the biopsy setting (Figures S6–S11), in our opinion, not acceptable for diagnostic tool. Importantly, there is no simple solution for such problems in weakly supervised training. On contrary, a supervised approach allows full flexibility of training, i.e., targeted extension of training dataset through problematic regions, balancing of classes, oversampling, custom batch creation, and other useful, often decisive techniques not accessible for weakly supervised approaches.

As a further downstream application of the developed computational platform, we suggest four new quantitative prognostic parameters to predict CSS, overall survival (OS), and PFS in LUAD and LUSC (Figures 6A–6C and S13), simple yet powerful and fully explainable. Such parameters cannot be reliably analyzed by human pathologists while the developed AI tool can quickly objectively calculate them.

Several published studies addressed the question of prognosis in patients with LUAD^36^^,^^37^ using deep learning (systematic review by Davri et al.^32^). All these studies use deep features from the convolutional networks which are not explainable, have very limited clinical use, and in general have moderate to low prognostic performance. All studies use small training datasets containing patient cases with and without recurrence (*n* = 55–393). The “hope” is that the algorithm itself finds the relevant discriminating features. The advantage of our developed parameters is their full explainability, biological sense, and applicability to both LUAD and LUSC. However, different thresholds might be necessary for these subtypes reflecting a differing biology and aggressivity (Figure 7D).

The favorable prognostic role of TLS was established in earlier studies for several cancer types including lung cancer.^38^^,^^39^ However, manual quantification might be difficult with high levels of interobserver variability.^39^ Two published studies approached TLS quantification and prognostic role using image analysis algorithms applied to H&E slides, one for LUAD^40^ and one for LUSC.^41^ Wang et al. showed the independent prognostic value of the higher absolute number of TLS in resectable LUAD.^40^ However, their tool uses regional classification algorithm principle, is not completely automatized, and requires manual intervention from pathologists for correction of TLS regions and filtering of small regions. van Rijthoven et al.^41^ developed a HookNet-based pixel-wise segmentation algorithm that detects only TLS in WSIs of LUSC cases without “seeing” the tissue context (requires some input from pathologists). The authors analyze only the prognostic role concerning OS. Like our results for this endpoint, they receive only marginal statistical significance (Figure S13). Our algorithm for quantification of TLS has several major advantages: (1) full automatization, (2) contextual analysis, (3) very high accuracy for TLS detection (Dice score 0.937), (4) applicability to both LUAD and LUSC subtypes, and (5) independent prognostic value additionally for PFS and CSS endpoints.

Moreover, we show that TLS can be effectively combined with one other new, fully quantitative, and explainable AI-derived parameter—namely necrosis density in tumor tissue (Figures 6B, 6C, and 7). This combination is intuitive while both parameters (TLS and necrosis density) mirror tumor biology/aggressiveness in two unique ways: TLS, containing the tumor by host immunity, and necrosis density, inherent/tumor cell aggressivity of tumor cells. Only a few small studies^42^^,^^43^^,^^44^ without image analysis assistance showed the prognostic value of tumor necrosis. All human-based quantifications are known for high levels of interobserver variability. To the best of our knowledge, we present the first AI-based algorithm for necrosis density quantification in lung cancer and show its independent prognostic value in LUAD (PFS, CSS, OS) and LUSC (CSS; PFS and OS—statistical trend) (Figures 7 and S13). Moreover, in combination with TLS (Figure 7), either as a direct T/NR ratio or using a cumulative prognostic grouping (T + NR), necrosis appears as a valuable marker for prognostic patient stratification in both LUAD and LUSC.

In conclusion, we developed a potent computational pathology platform for NSCLC. Using this platform, we trained an additional diagnostic algorithm for NSCLC subtyping and validated it using an international multi-institutional multi-scanner dataset. Moreover, we suggested four prognostic parameters and showed their strong capabilities to prognostically stratify NSCLC patients. The capabilities of progression prediction might be valuable for the selection of patients for adjuvant therapy after primary resection. We release four of our test datasets (including manually annotated test dataset) for academic research to allow further development, interoperability, and benchmarking of the published algorithms in the future.

### Limitations of the study

Our study is not devoid of limitations. The experiments performed are on the retrospective material, and prospective validation using multi-institutional cohorts is necessary. As our prognostic parameters are completely explainable and biologically intuitive, they are supposed to suffer substantially less from potential generalization problems compared to end-to-end deep learning models based on the “black box” deep features. Moreover, most AI-based pathology tools require continuous learning as there would be always out-of-distribution cases that were not present in the training dataset. Although our algorithm showed excellent generalization to unseen data, such situations could not be excluded.

## STAR★Methods

### Key resources table

**Table:** 

REAGENT or RESOURCE	SOURCE	IDENTIFIER
**Software and algorithms**		

Python 3.9.16	Conda	www.python.org
Pytorch 1.10 (training) and 2.0.1 (foundation models)	Pip	www.pytorch.org
Torchvision 0.15.2	Pip	www.pytorch.org
Numpy 1.24.3	Pip	www.numpy.org
Opencv-python 4.7.0.72	Pip	www.opencv.org
Pillow 9.5.0	Pip	www.pillow.readthedocs.io
Timm 0.4.12 (training) and 1.0.3 (foundation models)	Pip	https://github.com/huggingface/pytorch-image-models
Segmentation-model-pytorch 0.3.1	Pip	https://github.com/qubvel/segmentation_models.pytorch
Openslide 3.4.1	Conda	www.openslide.org
Openslide-python 1.2.0	Conda	www.openslide.org
Zarr 2.16.1	Pip	www.zarr.readthedocs.io
R 4.1.3	The R project for Statistical Computing	www.r-project.org
QuPath 0.4.3 and higher	QuPath	www.qupath.github.io
CLAM	GitHub	https://github.com/mahmoodlab/CLAM
UNI foundation model	Hugging Face	https://huggingface.co/MahmoodLab/UNI/
Prov-GigaPath foundation model	Hugging Face	https://huggingface.co/prov-gigapath/prov-gigapath/
Biorender	Biorender	www.biorender.com
Python & Groovy code	This study	https://github.com/cpath-ukk/lung_cancer Zenodo: https://doi.org/10.5281/zenodo.13132891
Checkpoints of CLAM models, UNI- and Prov-GigaPath-based classifiers	This study	Zenodo: 12818437

**Deposited data**		

Manually annotated dataset (UKK L1 SEGM)	This study	Zenodo: 12818382
Biopsy cases (WNS cohort), Part 1&2	This study	Zenodo: 12818177 & 12818228
Biopsy cases (UKK cohort), Part 1&2	This study	Zenodo: 12810956 & 12817969
Challenging cases cohort (subtyping), Biopsies	This study	Zenodo: 12810098
Challenging cases cohort (subtyping), Resections	This study	Zenodo: 12809470

### Resource availability

#### Lead contact

Further information and resource requests should be directly to and will be fulfilled by the lead contact, Yuri Tolkach (iurii.tolkach@uk-koeln.de).

#### Materials availability

This study did not generate new unique reagents.

#### Data and code availability

• Several fully anonymized datasets generated are released for academic research use only at Zenodo (s. key resources table).
• Code used in this study is available at https://github.com/cpath-ukk/lung_cancer. The doi at Zenodo is: https://doi.org/10.5281/zenodo.13132891.
• Any additional information required to reanalyze the data reported in this work paper is available from the lead contact upon request.

### Experimental model and subject details

#### Patient cases (training cohort)

The WSIs of the Cancer Genome Atlas (TCGA) lung cancer LUAD and LUSC cohorts were used for algorithm development. These slide images stem from 36 different pathology institutes and represent a highly heterogeneous cohort concerning tumor morphologies and lab practices (Figure 1B).

#### Patient cases (test cohort: Formal validation)

From one available external independent cohort, Institute of Pathology, University Hospital Cologne/Lab 1, resection specimens (UKK L1 RES), we selected a subset of 40 cases (LUAD *n* = 20, LUSC *n* = 20) representing a broad range of morphologies, patterns, and grades (including mucinous subtype of LUAD), UKK L1 SEGM (Figure 1B). These cases were annotated manually according to annotation principles and using the same tissue classes as for the training cohort.

#### Patient cases (training data: subtyping algorithm)

All available slides from LUAD and LUSC TCGA were included in the training (LUAD *n* = 531, LUSC *n* = 530) excluding slides with quality control issues.

#### Patient cases (test cohorts: subtyping algorithm validation)

Six independent cohorts of patient cases/WSI were included into clinical validation of subtyping algorithm with a total number of slides 4097 (LUAD *n* = 2723, LUSC *n* = 1374): UKK L1 RES, UKK Lab 2 biopsy cases (UKK L2 BX), University Hospital Aachen (Germany) – resection cases (AAC RES), Regional Hospital Wiener Neustadt (Austria) – resection cases (WNS RES) and biopsy cases (WNS BX), Kameda Hospital (Japan) – resection cases (KAM RES), University Hospital Essen (Germany) – Resection cases (ESS RES), and one public cohort from Clinical Proteomic Tumor Analysis Consortium (CPTAC, USA), including LUAD (CPTAC LUAD) and LUSC (CPTAC LUSC) cases. Details of digitization and the number of cases are provided in Figure 1B. The test cohorts were digitized by the five most common scanning systems. For biopsy cases, included were only tissue biopsies with cohesive tissue particles (transcutaneous needle and endobronchial biopsies), all cytological preparations or highly fragmented tissue without cohesive tissue parts were excluded. Also, deeper levels of the same biopsies were included to address the reproducibility of diagnosis.

#### Test cohorts (prognostic studies)

Entire LUAD (patients *n* = 446) and LUSC (patients *n* = 460) cohorts of TCGA with available follow-up data were used for the analysis of the prognostic value of new AI-derived parameters. Full clinicopathological characteristics of cohorts are provided in Table S1.

#### Ethics approval and consent to participate

All study steps were performed in accordance with the Declaration of Helsinki. This study was approved by the Ethical committee of the University of Cologne and University Hospital Essen (joint 22–1233, Project FED-PATH; Cologne 20–1583; Essen 13-5382-BO), the Ethical committee of Lower Austria (GS1-EK-4/694–2021), Kameda Hospital (22–094), University Hospital Aachen (EK 405/2). The need for patient consent was waived as only anonymized materials were used.

### Method details

#### Creation of training dataset: Multi-class tissue segmentatipn algorithm

The annotations were performed in QuPath software.^45^ Representative slide images were selected from the LUAD (*n* = 177) and LUSC (*n* = 107) cohorts with a full spectrum of morphologies and histological grades (Figure 1B). The slides were with high precision manually annotated by three experienced human analysts (YT, YW, CK), and reviewed/corrected by an experienced board-certified pathologist (YT). The principle of annotations is outlined in Figure 1D. Fourteen tissue classes were selected and annotated in all images. Later, for training purposes, four connective tissue classes (connective tissue/stroma, fatty tissue, muscle tissue, vessels with muscular wall) were merged. Background annotations were generated by algorithm developed earlier.^9^ The whole annotation process took approximately 18 months of work.

#### Model training: Multi-class tissue segmentation algorithm

All annotated regions were extracted in form of tiles with corresponding pixel maps. Algorithm training was performed in PyTorch framework v.1.10 and Python 3.9. Segmentation-models-pytorch library v.0.3.1 was used for neural network construction. A small subset of approximately 10% of images was reserved for fine-tuning (validation subset). Different hyperparameters were tested such as segmentation network, encoder backbone, tile extraction magnification, tile size, batch size, learning rate, and augmentation principles. All models were trained using oversampling of under-represented tissue classes and batch-level class balancing. Data augmentation was performed during training according to Tellez et al.2 (flips, rotations, brightness, contrast, gamma, hue, saturation). No stain normalization was used during training or later during inference. The algorithm is computationally lightweight and requires only 1.5 Gb of GPU RAM and can be highly parallelized on typical consumer grade GPUs (e.g., NVIDIA RTX3090). The final algorithm is based on the UNet++ decoder, EfficientNetB0 encoder (ImageNet pre-training; complete retraining on study dataset), works under μm/px (MMP) 1.0 (roughly 10x magnification), and uses a tile size of 512 px. The typical WSI analysis time for a resection case is 3–5 min and <1 min for a biopsy case (without parallelization). No additional filtering is performed after the WSI inference.

#### Creation of training dataset: Pixel wise-segmentation-based lung subtyping algorithm

Automatized annotation pipeline was constructed for slides other than already annotated manually in course of multi-class segmentation backbone algorithm development. The developed multi-class tissue segmentation algorithm allows for highly precise segmentation of all classes. Two to three representative rectangle regions with tumor tissue (of similar size as shown in Figure 1D) were manually selected by board-certified pathologist (YT) that represent intra-slide morphological tumor heterogeneity. These regions were extracted and segmented by the backbone algorithm. Annotations were created from segmentation masks and re-imported into annotation software. This allowed to include all TCGA cases into training. All annotation were checked for correctness. For all slides the quality of annotations/segmentation was excellent.

#### Model training: Pixel wise-segmentation-based lung subtyping algorithm

The algorithm was developed using similar principles as outlined above for the main backbone algorithm (Unet++/EfficientNetB0/MPP1.0/512 px). Six classes were used for training: tumor LUAD, tumor LUSC, tumor stroma, necrotic areas, mucin, and background. The classes other than tumor LUAD and tumor LUSC are primarily used to enhance the feature extraction capabilities of the algorithm.

#### Inference principle: Pixel wise-segmentation-based lung subtyping algorithm

The model is applied only to regions that were recognized as epithelial tumor component by the main algorithm. The inference map is a pixel-wise segmentation map visualized to pathologists and used for quantification of LUSC/LUAD areas within a tumor (percentage) which is used for final classification. Moreover, % of mucin (area mucin/area mucin + tumor + tumor stroma) is visualized to pathologists to decide the mucinous subtype of LUAD straightforwardly (Figure 4A).

#### Expert pathologists

Five expert board-certified pathologists regularly working with lung cancer cases were included in the clinical validation of the subtyping algorithm using a subset of “challenging” cases (RB, AQ, YT, YW, AS).

#### Implementation of subtyping algorithm using foundation models

Two foundation models: UNI (Chen et al. Nature Medicine 2024^18^) and Prov-Gigapath (Xu et al. Nature 2024^19^) that showed superior performance to “earlier” foundation models (e.g., CTransPath, REMEDIS, HIPT) but were not compared to each other, we used for experiments. The Prov-Gigapath model is based on the "vit_giant_patch14_dinov2″ architecture and produces a feature vector of length 1536; UNI uses "vit_large_patch16_224″ architecture resulting in feature vector of length 1024. Both work with image size of 224x224 px. Image transformation before feature extraction implements only native baseline normalization. The checkpoints were downloaded from hugging face repositories.

In this experiment, we explicitly evaluate the feature extraction capabilities without involving any additional (e.g., attention) methods. We use full subtyping dataset (FULL; using original train and validation split from supervised experiments) but also investigate an additional aspect and train on reduced amounts of available training data from our subtyping dataset (always accounting for case-level splits): 10%, 20%, 50%. The trainings on reduced amount of data are triplicates with three different, random case sets (e.g., 10%-v1, 10%-v2, 10%-v3). Importantly, for reduced amount of data, same subsets were used for UNI and Prov-GigaPath-based models (i.e., 10%-v1 split are the same cases for both models) for comparability. From original dataset with crop the patches to central regions of size 224x224 px using MPP 1.0 px/μm resolution.

Two layers were added to the model for training – one fully connected layer with 512 parameters and one final classification layer with one parameter and sigmoid activation. In each training subset the model was trained for maximal 100 epochs and best checkpoint was selected based on the overall accuracy on the validation subset. All trainings were performed using batch size 8 (batch size of 16 was also implemented but showed slightly worse results). Random oversampling was used to extend the slightly underrepresented class (LUAD) to match the number of patches in LUSC class.

Patch-level validation was implemented on the UKK L1 SEGM dataset (only tumor containing patches with subtype label; extraction without overlap, patch size 224x224 px, MPP 1.0). Case-level validation was used using original pipeline for segmentation whereby the subtyping model was replaced by trained foundation extraction-based classification module.

#### Implementation of subtyping algorithm using CLAM and UNI encoder

Original implementation of CLAM^23^ was used available from https://github.com/mahmoodlab/CLAM. To allow comparability with fully supervised segmentation-based approach and feature extraction-based approach described above, the training was performed using TCGA dataset in original splits using 10-folds to account for intra-dataset heterogeneities and batch effects. The patch extraction was performed at MPP 1.0 with patch size of 224px without overlap. The feature extraction was performed using UNI foundational encoder. The tests were performed on the independent test datasets on the slide level. All visualization steps were performed using original pipeline. All heatmaps were evaluated by experiences, board certified pathologists. AUC metrics for comparison with earlier approaches were generated for each of 10-folds.

#### Quantification of prognostic parameters

Tertiary lymphoid structures (TLS) and necrosis quantification are based on the segmentation maps derived from the slide processing by the main multi-class tissue segmentation backbone algorithm (Figures 6A and 6B). We do not apply any forms of filtering. The necrosis area in a slide is measured as px2. Necrosis tumor density (NECR-TD) is quantified as “area necrosis”/“area tumor + area stroma”, at that “area necrosis” is a sum of all tumor-associated necrosis objects. TLS tumor density (TLS-TD) is quantified as “TLS area”/“area tumor + area stroma”. We quantify intratumoral and immediate peritumoral TLS objects. Optimal cut-off is used for dichotomization of the cohort cases into low and high groups (stratification with best prognostic significance). TLS/Necrosis (T/NR) area is quantified as a simple ratio between areas of these two types of objects, also dichotomized using the optimal cutoff. No additional normalization (e.g., using tumor area) is necessary in this case. Cumulative parameter is being built using dichotomized TLS-TD and NECR-TD parameters (T + NR), resulting in three prognostic groups (PG1-3; Figure 6C).

#### Hardware

The model training and tests were performed on the high-performance cluster of the University of Cologne equipped with 12x NVIDIA V100 32Gb graphic cards and on the AI server with 4x A100 NVIDIA cards (training, validation) as well on the PC stations equipped with NVIDIA RTX 3090/4090 graphic cards.

### Quantification and statistical analysis

All statistical tests were carried out in R v. 4.1.3 (The R Foundation for Statistical Computing). Typical accuracy metrics were calculated at different experimental stages: Dice score, specificity, sensitivity, overall accuracy, and F1 score. Kaplan Meier estimates with log rank test and univariate and multivariate Cox proportional hazards regression models were used for evaluation of the prognostic role of developed parameters. A receiver operating characteristic (ROC) curve analysis and an AUC analysis were done with 95% CI calculations and bootstrapping with 2000 bootstrap replicates (pROC package in R).

## Supplemental information

Document S1. Figures S1–S13 and Tables S1–S7

Document S2. Article plus supplemental information

## References

[1] Siegel R.L., Miller K.D., Wagle N.S., Jemal A. (2023). Cancer statistics, 2023. CA. Cancer J. Clin..

[2] Zhang Y., Vaccarella S., Morgan E., Li M., Etxeberria J., Chokunonga E., Manraj S.S., Kamate B., Omonisi A., Bray F. (2023). Global variations in lung cancer incidence by histological subtype in 2020: a population-based study. Lancet Oncol..

[3] Bera K., Schalper K.A., Rimm D.L., Velcheti V., Madabhushi A. (2019). Artificial intelligence in digital pathology — new tools for diagnosis and precision oncology. Nat. Rev. Clin. Oncol..

[4] Echle A., Rindtorff N.T., Brinker T.J., Luedde T., Pearson A.T., Kather J.N. (2021). Deep learning in cancer pathology: a new generation of clinical biomarkers. Br. J. Cancer.

[5] El Nahhas O.S.M., Loeffler C.M.L., Carrero Z.I., van Treeck M., Kolbinger F.R., Hewitt K.J., Muti H.S., Graziani M., Zeng Q., Calderaro J. (2024). Regression-based Deep-Learning predicts molecular biomarkers from pathology slides. Nat. Commun..

[6] Perez-Lopez R., Ghaffari L.N., Mahmood F., Kather J.N. (2024). A guide to artificial intelligence for cancer researchers. Nat. Rev. Cancer.

[7] Kleppe A., Skrede O.J., De Raedt S., Liestøl K., Kerr D.J., Danielsen H.E. (2021). Designing deep learning studies in cancer diagnostics. Nat. Rev. Cancer.

[8] Campanella G., Hanna M.G., Geneslaw L., Miraflor A., Werneck Krauss Silva V., Busam K.J., Brogi E., Reuter V.E., Klimstra D.S., Fuchs T.J. (2019). Clinical-grade computational pathology using weakly supervised deep learning on whole slide images. Nat. Med..

[9] Griem J., Eich M.-L., Schallenberg S., Pryalukhin A., Bychkov A., Fukuoka J., Zayats V., Hulla W., Munkhdelger J., Seper A. (2023). Artificial Intelligence-Based Tool for Tumor Detection and Quantitative Tissue Analysis in Colorectal Specimens. Mod. Pathol..

[10] Tolkach Y., Wolgast L.M., Damanakis A., Pryalukhin A., Schallenberg S., Hulla W., Eich M.L., Schroeder W., Mukhopadhyay A., Fuchs M. (2023). Artificial intelligence for tumour tissue detection and histological regression grading in oesophageal adenocarcinomas: a retrospective algorithm development and validation study. Lancet. Digit. Health.

[11] Tolkach Y., Ovtcharov V., Pryalukhin A., Eich M.L., Gaisa N.T., Braun M., Radzhabov A., Quaas A., Hammerer P., Dellmann A. (2023). An international multi-institutional validation study of the algorithm for prostate cancer detection and Gleason grading. NPJ Precis. Oncol..

[12] Ehteshami Bejnordi B., Veta M., Johannes van Diest P., van Ginneken B., Karssemeijer N., Litjens G., van der Laak J.A.W.M., Hermsen M., Manson Q.F., the CAMELYON16 Consortium (2017). Diagnostic Assessment of Deep Learning Algorithms for Detection of Lymph Node Metastases in Women With Breast Cancer. JAMA.

[13] Klein S., Quaas A., Quantius J., Löser H., Meinel J., Peifer M., Wagner S., Gattenlöhner S., Wittekindt C., von Knebel Doeberitz M. (2021). Deep Learning Predicts HPV Association in Oropharyngeal Squamous Cell Carcinomas and Identifies Patients with a Favorable Prognosis Using Regular H&E Stains. Clin. Cancer Res..

[14] Pantanowitz L., Quiroga-Garza G.M., Bien L., Heled R., Laifenfeld D., Linhart C., Sandbank J., Albrecht Shach A., Shalev V., Vecsler M. (2020). An artificial intelligence algorithm for prostate cancer diagnosis in whole slide images of core needle biopsies: a blinded clinical validation and deployment study. Lancet. Digit. Health.

[15] Campanella G., Ho D., Häggström I., Becker A.S., Chang J., Vanderbilt C., Fuchs T.J. (2022). H&E-based Computational Biomarker Enables Universal EGFR Screening for Lung Adenocarcinoma. arXiv.

[16] Schömig-Markiefka B., Pryalukhin A., Hulla W., Bychkov A., Fukuoka J., Madabhushi A., Achter V., Nieroda L., Büttner R., Quaas A., Tolkach Y. (2021). Quality control stress test for deep learning-based diagnostic model in digital pathology. Mod. Pathol..

[17] Lu M.Y., Chen B., Williamson D.F.K., Chen R.J., Liang I., Ding T., Jaume G., Odintsov I., Le L.P., Gerber G. (2024). A visual-language foundation model for computational pathology. Nat. Med..

[18] Chen R.J., Ding T., Lu M.Y., Williamson D.F.K., Jaume G., Song A.H., Chen B., Zhang A., Shao D., Shaban M. (2024). Towards a general-purpose foundation model for computational pathology. Nat. Med..

[19] Xu H., Usuyama N., Bagga J., Zhang S., Rao R., Naumann T., Wong C., Gero Z., González J., Gu Y. (2024). A whole-slide foundation model for digital pathology from real-world data. Nature.

[20] Wang X., Yang S., Zhang J., Wang M., Zhang J., Yang W., Huang J., Han X. (2022). Transformer-based unsupervised contrastive learning for histopathological image classification. Med. Image Anal..

[21] Ligero M., Serna G., El Nahhas O.S.M., Sansano I., Mauchanski S., Viaplana C., Calderaro J., Toledo R.A., Dienstmann R., Vanguri R.S. (2024). Weakly Supervised Deep Learning Predicts Immunotherapy Response in Solid Tumors Based on PD-L1 Expression. Cancer Res. Commun..

[22] Jiang X., Hoffmeister M., Brenner H., Muti H.S., Yuan T., Foersch S., West N.P., Brobeil A., Jonnagaddala J., Hawkins N. (2024). End-to-end prognostication in colorectal cancer by deep learning: a retrospective, multicentre study. Lancet. Digit. Health.

[23] Lu M.Y., Williamson D.F.K., Chen T.Y., Chen R.J., Barbieri M., Mahmood F. (2021). Data-efficient and weakly supervised computational pathology on whole-slide images. Nat. Biomed. Eng..

[24] Lipkova J., Chen R.J., Chen B., Lu M.Y., Barbieri M., Shao D., Vaidya A.J., Chen C., Zhuang L., Williamson D.F.K. (2022). Artificial intelligence for multimodal data integration in oncology. Cancer Cell.

[25] Gilbert S., Kather J.N. (2024). Guardrails for the use of generalist AI in cancer care. Nat. Rev. Cancer.

[26] Jain D.K., Lakshmi K.M., Varma K.P., Ramachandran M., Bharati S. (2022). Lung Cancer Detection Based on Kernel PCA-Convolution Neural Network Feature Extraction and Classification by Fast Deep Belief Neural Network in Disease Management Using Multimedia Data Sources. Comput. Intell. Neurosci..

[27] Rączkowski Ł., Paśnik I., Kukiełka M., Nicoś M., Budzinska M.A., Kucharczyk T., Szumiło J., Krawczyk P., Crosetto N., Szczurek E. (2022). Deep learning-based tumor microenvironment segmentation is predictive of tumor mutations and patient survival in non-small-cell lung cancer. BMC Cancer.

[28] Anjum S., Ahmed I., Asif M., Aljuaid H., Alturise F., Ghadi Y.Y., Elhabob R. (2023). Lung Cancer Classification in Histopathology Images Using Multiresolution Efficient Nets. Comput. Intell. Neurosci..

[29] Kriegsmann M., Haag C., Weis C.A., Steinbuss G., Warth A., Zgorzelski C., Muley T., Winter H., Eichhorn M., Eichhorn F. (2020). Deep Learning for the Classification of Small-Cell and Non-Small-Cell Lung Cancer. Cancers.

[30] Li Z., Zhang J., Tan T., Teng X., Sun X., Zhao H., Liu L., Xiao Y., Lee B., Li Y. (2021). Deep Learning Methods for Lung Cancer Segmentation in Whole-Slide Histopathology Images-The ACDC@LungHP Challenge 2019. IEEE J. Biomed. Health Inform..

[31] Yang H., Chen L., Cheng Z., Yang M., Wang J., Lin C., Wang Y., Huang L., Chen Y., Peng S. (2021). Deep learning-based six-type classifier for lung cancer and mimics from histopathological whole slide images: a retrospective study. BMC Med..

[32] Davri A., Birbas E., Kanavos T., Ntritsos G., Giannakeas N., Tzallas A.T., Batistatou A. (2023). Deep Learning for Lung Cancer Diagnosis, Prognosis and Prediction Using Histological and Cytological Images: A Systematic Review. Cancers.

[33] Chen C.L., Chen C.C., Yu W.H., Chen S.H., Chang Y.C., Hsu T.I., Hsiao M., Yeh C.Y., Chen C.Y. (2021). An annotation-free whole-slide training approach to pathological classification of lung cancer types using deep learning. Nat. Commun..

[34] Dolezal J.M., Srisuwananukorn A., Karpeyev D., Ramesh S., Kochanny S., Cody B., Mansfield A.S., Rakshit S., Bansal R., Bois M.C. (2022). Uncertainty-informed deep learning models enable high-confidence predictions for digital histopathology. Nat. Commun..

[35] Coudray N., Ocampo P.S., Sakellaropoulos T., Narula N., Snuderl M., Fenyö D., Moreira A.L., Razavian N., Tsirigos A. (2018). Classification and mutation prediction from non-small cell lung cancer histopathology images using deep learning. Nat. Med..

[36] Qaiser T., Lee C.Y., Vandenberghe M., Yeh J., Gavrielides M.A., Hipp J., Scott M., Reischl J. (2022). Usability of deep learning and H&E images predict disease outcome-emerging tool to optimize clinical trials. npj Precis. Oncol..

[37] Wu Z., Wang L., Li C., Cai Y., Liang Y., Mo X., Lu Q., Dong L., Liu Y. (2020). DeepLRHE: A Deep Convolutional Neural Network Framework to Evaluate the Risk of Lung Cancer Recurrence and Metastasis From Histopathology Images. Front. Genet..

[38] Silina K., Soltermann A., Attar F.M., Casanova R., Uckeley Z.M., Thut H., Wandres M., Isajevs S., Cheng P., Curioni-Fontecedro A. (2018). Germinal centers determine the prognostic relevance of tertiary lymphoid structures and are impaired by corticosteroids in lung squamous cell carcinoma. Cancer Res..

[39] Rakaee M., Kilvaer T.K., Jamaly S., Berg T., Paulsen E.E., Berglund M., Richardsen E., Andersen S., Al-Saad S., Poehl M. (2021). Tertiary lymphoid structure score: a promising approach to refine the TNM staging in resected non-small cell lung cancer. Br. J. Cancer.

[40] Wang Y., Lin H., Yao N., Chen X., Qiu B., Cui Y., Liu Y., Li B., Han C., Li Z. (2023). Computerized tertiary lymphoid structures density on H&E-images is a prognostic biomarker in resectable lung adenocarcinoma. iScience.

[41] van Rijthoven M., Obahor S., Pagliarulo F., van den Broek M., Schraml P., Moch H., van der Laak J., Ciompi F., Silina K. (2024). Multi-resolution deep learning characterizes tertiary lymphoid structures and their prognostic relevance in solid tumors. Commun. Med..

[42] Park S.Y., Lee H.S., Jang H.J., Lee G.K., Chung K.Y., Zo J.I. (2011). Tumor necrosis as a prognostic factor for stage IA non-small cell lung cancer. Ann. Thorac. Surg..

[43] Moon S.W., Kim J.J., Jeong S.C., Kim Y.H., Han J.W. (2022). Clinical significance of tumor necrosis and viability in non-small cell lung cancer. J. Thorac. Dis..

[44] Swinson D.E.B., Jones J.L., Richardson D., Cox G., Edwards J.G., O’Byrne K.J. (2002). Tumour necrosis is an independent prognostic marker in non-small cell lung cancer: Correlation with biological variables. Lung Cancer.

[45] Bankhead P., Loughrey M.B., Fernández J.A., Dombrowski Y., McArt D.G., Dunne P.D., McQuaid S., Gray R.T., Murray L.J., Coleman H.G. (2017). QuPath: Open source software for digital pathology image analysis. Sci. Rep..

