# Supplementary material for: Next-generation lung cancer pathology: Development and validation of diagnostic and prognostic algorithms
Source: Cell Rep Med. 2024 Aug 22;5(9):101697. doi: 10.1016/j.xcrm.2024.101697 (PMC11524894; doi:10.1016/j.xcrm.2024.101697)
Supplement: Document S1. Figures S1–S13 and Tables S1–S7 [file mmc1.pdf]

**Cell Reports Medicine, Volume *5***

## Supplemental information

# Next-generation lung cancer pathology: Development and validation of diagnostic and prognostic algorithms

**Carina Kludt, Yuan Wang, Waleed Ahmad, Andrey Bychkov, Junya Fukuoka, Nadine Gaisa, Mark Kühnel, Danny Jonigk, Alexey Pryalukhin, Fabian Mairinger, Franziska Klein, Anne Maria Schultheis, Alexander Seper, Wolfgang Hulla, Johannes Brägelmann, Sebastian Michels, Sebastian Klein, Alexander Quaas, Reinhard Büttner, and Yuri Tolkach**

**Supplementary information**

**Next generation lung cancer pathology:**

**development and validation of diagnostic and prognostic algorithms**

Carina Kludt, Yuan Wang, Waleed Ahmad, Andrey Bychkov, Junya Fukuoka, Nadine Gaisa, Mark Kühnel, Danny Jonigk, Alexey Pryalukhin, Fabian Mairinger, Franziska Klein, Anne Maria Schultheis, Alexander Seper, Wolfgang Hulla, Johannes Brägelmann, Sebastian Michels, Sebastian Klein, Alexander Quaas, Reinhard Büttner, Yuri Tolkach

**Table S1 Clinicopathological characteristics of the study cohorts (prognostic analysis). Related to Figures 1 and 7**

| Parameter | LUAD cohort (n=446) | | LUSC cohort (n=460) | |
|---|---|---|---|---|
| | n | % | n | % |
| **Sex** | | | | |
| female | 239 | 53.6 | 118 | 25.7 |
| male | 207 | 46.4 | 342 | 74.3 |
| **pT stage** | | | | |
| pT1 | 155 | 34.8 | 103 | 22.4 |
| pT2 | 233 | 52.2 | 273 | 59.3 |
| pT3 | 39 | 8.7 | 63 | 13.7 |
| pT4 | 16 | 3.6 | 21 | 4.6 |
| unknown | 3 | 0.7 | 0 | 0 |
| **pN stage** | | | | |
| pN0 | 294 | 65.9 | 291 | 63.3 |
| pN1 | 87 | 19.5 | 123 | 26.7 |
| pN2 | 55 | 12.3 | 41 | 8.9 |
| unknown | 10 | 2.2 | 5 | 1.1 |
| **UICC stage** | | | | |
| I | 244 | 54.7 | 225 | 48.9 |
| II | 108 | 24.2 | 148 | 32.2 |
| III | 61 | 13.7 | 77 | 16.7 |
| IV | 25 | 5.6 | 6 | 1.3 |
| unknown | 8 | 1.8 | 4 | 0.9 |
| **OS** | | | | |
| Alive | 289 | 64.8 | 264 | 57.4 |
| Deceased | 157 | 35.2 | 196 | 42.6 |
| Not available | - | | - | |
| **CSS** | | | | |
| Alive | 315 | 70.6 | 314 | 68.3 |
| Deceased | 90 | 20.2 | 102 | 22.2 |
| Not available | 41 | 9.2 | 44 | 9.6 |
| **PFS** | | | | |
| Progression | 116 | 26.0 | 111 | 24.1 |
| Censored | 255 | 57.2 | 281 | 61.1 |
| Not available | 75 | 16.8 | 68 | 14.8 |
| **Follow-up duration** | | | | |
| Mean (SD), months | 31.2 (29.6) | | 34 (32.3) | |
| Range, months | 1-242 | | 1-177 | |
| **Age** | | | | |
| Min | 33 | | 39 | |
| Max | 88 | | 90 | |
| Mean | 65.25 | | 67.3 | |
| SD | 10.2 | | 8.6 | |
| **Smoking history** | | | | |
| Non-smoker | 61 | 13.7% | 14 | 3.0% |
| Smoker | 379 | 85.0% | 438 | 95.2% |
| Not available | 6 | 1.3% | 8 | 1.8% |

Comment: SD – standard deviation

**Table S2 Results of univariate Cox regression analysis in lung adenocarcinoma cohort. Related to Figures 6 and 7**

| | | | | |
|---|---|---|---|---|
| **TLS-TD** | **CSS** | | | |
| | HR | 95%CI lower | 95%CI upper | p-value |
| high | 1 | | | |
| low | 1.65 | 0.99 | 2.85 | 0.055 |
| **TLS-TD** | **OS** | | | |
| | HR | 95%CI lower | 95%CI upper | p-value |
| high | 1 | | | |
| low | 1.69 | 1.12 | 2.53 | 0.012 |
| **TLS-TD** | **PFS** | | | |
| | HR | 95%CI lower | 95%CI upper | p-value |
| high | 1 | | | |
| low | 2.04 | 1.3 | 3.2 | 0.002 |
| **NECR-TD** | **CSS** | | | |
| | HR | 95%CI lower | 95%CI upper | p-value |
| low | 1 | | | |
| high | 2.44 | 1.23 | 4.86 | 0.011 |
| **NECR-TD** | **OS** | | | |
| | HR | 95%CI lower | 95%CI upper | p-value |
| low | 1 | | | |
| high | 1.88 | 1.17 | 3.04 | 0.0097 |
| **NECR-TD** | **PFS** | | | |
| | HR | 95%CI lower | 95%CI upper | p-value |
| low | 1 | | | |
| high | 2.56 | 1.41 | 4.66 | 0.002 |
| **T/NR** | CSS | | | |
| | HR | 95%CI lower | 95%CI upper | p-value |
| low | 1 | | | |
| high | 1.99 | 1.31 | 3.04 | 0.001 |
| **T/NR** | OS | | | |
| | HR | 95%CI lower | 95%CI upper | p-value |
| low | 1 | | | |
| high | 1.66 | 1.2 | 2.3 | 0.002 |

| **T/NR** | PFS | | | |
|---|---|---|---|---|
| | HR | 95%CI lower | 95%CI upper | p-value |
| low | 1 | | | |
| high | 2.2 | 1.52 | 3.19 | 2.90E-05 |

| **TLS-TD + NECR-TD** | **CSS** | | | |
|---|---|---|---|---|
| | HR | 95%CI lower | 95%CI upper | p-value |
| T high, N low | 1 | | | |
| T high + N high / T low + N low | 2.53 | 1.16 | 5.5 | 0.019 |
| T low + N high | 4.21 | 1.71 | 10.36 | 0.002 |

| **TLS-TD + NECR-TD** | **OS** | | | |
|---|---|---|---|---|
| | HR | 95%CI lower | 95%CI upper | p-value |
| T high, N low | 1 | | | |
| T high + N high / T low + N low | 1.96 | 1.14 | 3.35 | 0.015 |
| T low + N high | 3.22 | 1.7 | 6.13 | 0.0004 |

| **TLS-TD + NECR-TD** | **PFS** | | | |
|---|---|---|---|---|
| | HR | 95%CI lower | 95%CI upper | p-value |
| T high, N low | 1 | | | |
| T high + N high / T low + N low | 2.72 | 1.37 | 5.4 | 0.004 |
| T low + N high | 5.41 | 2.47 | 11.84 | 2.40E-05 |

**Table S3 Results of univariate Cox regression analysis in lung squamous cell carcinoma cohort. Related to Figures 6 and 7**

| **TLS-TD** | **CSS** | | | |
|---|---|---|---|---|
| | HR | 95%CI lower | 95%CI upper | p-value |
| high | 1 | | | |
| low | 1.93 | 1.15 | 3.25 | 0.013 |

| **TLS-TD** | **OS** | | | |
|---|---|---|---|---|
| | HR | 95%CI lower | 95%CI upper | p-value |
| high | 1 | | | |
| low | 1.22 | 0.88 | 1.7 | 0.229 |

| **TLS-TD** | **PFS** | | | |
|---|---|---|---|---|
| | HR | 95%CI lower | 95%CI upper | p-value |
| high | 1 | | | |
| low | 1.91 | 1.17 | 3.09 | 0.009 |

| **NECR-TD** | **CSS** | | | |
|---|---|---|---|---|
| | HR | 95%CI lower | 95%CI upper | p-value |
| low | 1 | | | |
| high | 1.71 | 1.12 | 2.59 | 0.013 |

| **NECR-TD** | **OS** | | | |
|---|---|---|---|---|
| | HR | 95%CI lower | 95%CI upper | p-value |
| low | 1 | | | |
| high | 1.32 | 0.99 | 1.77 | 0.063 |

| **NECR-TD** | **PFS** | | | |
|---|---|---|---|---|
| | HR | 95%CI lower | 95%CI upper | p-value |
| low | 1 | | | |
| high | 1.44 | 0.97 | 2.13 | 0.071 |

| **T/NR** | CSS | | | |
|---|---|---|---|---|
| | HR | 95%CI lower | 95%CI upper | p-value |
| low | 1 | | | |
| high | 2.48 | 1.49 | 4.13 | 0.0005 |

| **T/NR** | OS | | | |
|---|---|---|---|---|
| | HR | 95%CI lower | 95%CI upper | p-value |
| low | 1 | | | |
| high | 1.47 | 1.07 | 2.02 | 0.018 |

| **T/NR** | PFS | | | |
|---|---|---|---|---|

| | HR | 95%CI lower | 95%CI upper | p-value |
|---|---|---|---|---|
| low | 1 | | | |
| high | 1.98 | 1.26 | 3.11 | 0.003 |

| **TLS-TD + NECR-TD** | **CSS** | | | |
|---|---|---|---|---|
| | HR | 95%CI lower | 95%CI upper | p-value |
| T high, N low | 1 | | | |
| T high + N high / T low + N low | 2 | 0.85 | 4.74 | 0.11 |
| T low + N high | 3.27 | 1.41 | 7.58 | 0.006 |

| **TLS-TD + NECR-TD** | **OS** | | | |
|---|---|---|---|---|
| | HR | 95%CI lower | 95%CI upper | p-value |
| T high, N low | 1 | | | |
| T high + N high / T low + N low | 1.3 | 0.79 | 2.13 | 0.305 |
| T low + N high | 1.6 | 0.98 | 2.61 | 0.058 |

| **TLS-TD + NECR-TD** | **PFS** | | | |
|---|---|---|---|---|
| | HR | 95%CI lower | 95%CI upper | p-value |
| T high, N low | 1 | | | |
| T high + N high / T low + N low | 2.02 | 0.91 | 4.49 | 0.084 |
| T low + N high | 2.9 | 1.32 | 6.34 | 0.008 |

**Table S4 Results of multivariate Cox regression analysis in lung adenocarcinoma cohort. Related to Figures 6 and 7**

| **TLS-TD** | **CSS** | | | |
|---|---|---|---|---|
| | HR | 95%CI lower | 95%CI upper | p-value |
| high | 1 | | | |
| low | 1.7 | 0.99 | 2.91 | 0.053 |
| pT1 | 1 | | | |
| pT2 | 1.07 | 0.64 | 1.78 | 0.81 |
| pT3 | 3.07 | 1.53 | 6.15 | 0.0016 |
| pT4 | 3.85 | 1.41 | 10.53 | 0.009 |
| pN0 | 1 | | | |
| pN1 | 2.36 | 1.45 | 3.84 | 0.0005 |
| pN2 | 2.16 | 1.17 | 3.97 | 0.014 |

| **TLS-TD** | **OS** | | | |
|---|---|---|---|---|
| | HR | 95%CI lower | 95%CI upper | p-value |
| high | 1 | | | |
| low | 1.52 | 1 | 2.31 | 0.052 |
| pT1 | 1 | | | |
| pT2 | 1.15 | 0.78 | 1.7 | 0.481 |
| pT3 | 2.73 | 1.57 | 4.76 | 0.0004 |
| pT4 | 2.32 | 1.13 | 4.76 | 0.021 |
| pN0 | 1 | | | |
| pN1 | 2.22 | 1.53 | 3.21 | 2.50E-05 |
| pN2 | 2.56 | 1.63 | 4 | 4.02E-05 |

| **TLS-TD** | **PFS** | | | |
|---|---|---|---|---|
| | HR | 95%CI lower | 95%CI upper | p-value |
| high | 1 | | | |
| low | 1.91 | 1.21 | 3.05 | 0.006 |
| pT1 | 1 | | | |
| pT2 | 1.46 | 0.94 | 2.27 | 0.091 |
| pT3 | 3.44 | 1.84 | 6.43 | 0.0001 |
| pT4 | 2.17 | 0.75 | 6.25 | 0.151 |
| pN0 | 1 | | | |
| pN1 | 1.77 | 1.15 | 2.73 | 0.01 |
| pN2 | 1.73 | 0.98 | 3.05 | 0.06 |

| **NECR-TD** | **CSS** | | | |
|---|---|---|---|---|
| | HR | 95%CI lower | 95%CI upper | p-value |
| high | 1 | | | |
| low | 2.24 | 1.12 | 4.49 | 0.022 |
| pT1 | 1 | | | |
| pT2 | 1.02 | 0.61 | 1.71 | 0.93 |
| pT3 | 3.11 | 1.56 | 6.25 | 0.001 |
| pT4 | 3.78 | 1.38 | 10.37 | 0.009 |
| pN0 | 1 | | | |
| pN1 | 2.27 | 1.4 | 3.68 | 0.0009 |
| pN2 | 2.1 | 1.14 | 3.87 | 0.018 |

| **NECR-TD** | **OS** | | | |
|---|---|---|---|---|
| | HR | 95%CI lower | 95%CI upper | p-value |
| high | 1 | | | |
| low | 1.71 | 1.06 | 2.79 | 0.029 |
| pT1 | 1 | | | |
| pT2 | 1.1 | 0.74 | 1.62 | 0.645 |
| pT3 | 2.74 | 1.58 | 4.78 | 0.0004 |
| pT4 | 2.34 | 1.15 | 4.76 | 0.019 |
| pN0 | 1 | | | |
| pN1 | 2.21 | 1.53 | 3.2 | 2.40E-05 |
| pN2 | 2.53 | 1.61 | 3.96 | 5.16E-05 |

| **NECR-TD** | **PFS** | | | |
|---|---|---|---|---|
| | HR | 95%CI lower | 95%CI upper | p-value |
| high | 1 | | | |
| low | 2.33 | 1.27 | 4.28 | 0.006 |
| pT1 | 1 | | | |
| pT2 | 1.34 | 0.86 | 2.09 | 0.191 |
| pT3 | 3.7 | 1.98 | 6.93 | 4.22E-05 |
| pT4 | 2.1 | 0.73 | 6.05 | 0.17 |
| pN0 | 1 | | | |
| pN1 | 1.67 | 1.08 | 2.57 | 0.021 |
| pN2 | 1.56 | 0.88 | 2.76 | 0.124 |

| **T/NR** | CSS | | | |
|---|---|---|---|---|
| | HR | 95%CI lower | 95%CI upper | p-value |
| high | 1 | | | |
| low | 2.04 | 1.33 | 3.13 | 0.001 |
| pT1 | 1 | | | |
| pT2 | 1.05 | 0.63 | 1.75 | 0.846 |
| pT3 | 3.12 | 1.56 | 6.25 | 0.001 |
| pT4 | 4.27 | 1.58 | 11.57 | 0.004 |
| pN0 | 1 | | | |
| pN1 | 2.38 | 1.46 | 3.85 | 0.0005 |
| pN2 | 2.08 | 1.14 | 3.81 | 0.017 |

| **T/NR** | OS | | | |
|---|---|---|---|---|
| | HR | 95%CI lower | 95%CI upper | p-value |
| high | 1 | | | |
| low | 1.64 | 1.18 | 2.28 | 0.004 |
| pT1 | 1 | | | |
| pT2 | 1.14 | 0.78 | 1.68 | 0.501 |
| pT3 | 2.74 | 1.58 | 4.77 | 0.0003 |
| pT4 | 2.66 | 1.31 | 5.4 | 0.007 |
| pN0 | 1 | | | |
| pN1 | 2.25 | 1.55 | 3.25 | 1.75E-05 |
| pN2 | 2.47 | 1.58 | 2.28 | 7.08E-05 |

| T/NR | PFS | | | |
|---|---|---|---|---|
| | HR | 95%CI lower | 95%CI upper | p-value |
| high | 1 | | | |
| low | 2.06 | 1.41 | 3 | 0.0002 |
| pT1 | 1 | | | |
| pT2 | 1.42 | 0.92 | 2.2 | 0.112 |
| pT3 | 3.52 | 1.89 | 6.59 | 7.47E-05 |
| pT4 | 2.51 | 0.87 | 7.19 | 0.087 |
| pN0 | 1 | | | |
| pN1 | 1.79 | 1.16 | 2.76 | 0.008 |
| pN2 | 1.51 | 0.86 | 2.66 | 0.153 |

| **TLS-TD + NECR-TD** | **CSS** | | | |
|---|---|---|---|---|
| | HR | 95%CI lower | 95%CI upper | p-value |
| T high, N low | 1 | | | |
| T high + N high / T low + N low | 2.32 | 1.06 | 5.08 | 0.035 |
| T low + N high | 3.98 | 1.59 | 9.93 | 0.003 |
| pT1 | 1 | | | |
| pT2 | 0.99 | 0.59 | 1.66 | 0.973 |
| pT3 | 3.01 | 1.5 | 6.05 | 0.002 |
| pT4 | 3.38 | 1.22 | 9.37 | 0.019 |
| pN0 | 1 | | | |
| pN1 | 2.33 | 1.43 | 3.79 | 0.0007 |
| pN2 | 2.23 | 1.21 | 4.13 | 0.01 |

| **TLS-TD + NECR-TD** | **OS** | | | |
|---|---|---|---|---|
| | HR | 95%CI lower | 95%CI upper | p-value |
| T high, N low | 1 | | | |
| T high + N high / T low + N low | 1.8 | 1.05 | 3.1 | 0.033 |
| T low + N high | 2.68 | 1.38 | 5.2 | 0.004 |
| pT1 | 1 | | | |
| pT2 | 1.08 | 0.73 | 1.59 | 0.717 |
| pT3 | 2.65 | 1.52 | 4.63 | 0.0006 |
| pT4 | 2.1 | 1.02 | 4.34 | 0.044 |
| pN0 | 1 | | | |
| pN1 | 2.21 | 1.52 | 3.2 | 2.90E-05 |
| pN2 | 2.59 | 1.66 | 4.06 | 3.13E-05 |

| **TLS-TD + NECR-TD** | **PFS** | | | |
|---|---|---|---|---|
| | HR | 95%CI lower | 95%CI upper | p-value |
| T high, N low | 1 | | | |
| T high + N high / T low + N low | 2.5 | 1.25 | 4.99 | 0.0096 |
| T low + N high | 4.66 | 2.09 | 10.41 | 0.00017 |
| pT1 | 1 | | | |
| pT2 | 1.28 | 0.82 | 2 | 0.273 |
| pT3 | 3.42 | 1.82 | 6.42 | 0.0001 |
| pT4 | 1.94 | 0.67 | 5.61 | 0.224 |
| pN0 | 1 | | | |
| pN1 | 1.73 | 1.12 | 2.68 | 0.013 |
| pN2 | 1.69 | 0.95 | 2.99 | 0.073 |

**Table S5 Results of multivariate Cox regression analysis in lung squamous cell carcinoma cohort. Related to Figures 6 and 7**

| **TLS-TD** | **CSS** | | | |
|---|---|---|---|---|
| | HR | 95%CI lower | 95%CI upper | p-value |
| high | 1 | | | |
| low | 1.9 | 1.12 | 3.23 | 0.018 |
| pT1 | 1 | | | |
| pT2 | 1.19 | 0.7 | 2.02 | 0.526 |
| pT3 | 2 | 1.03 | 3.86 | 0.04 |
| pT4 | 3.44 | 1.45 | 8.15 | 0.005 |
| pN0 | 1 | | | |
| pN1 | 1.32 | 0.84 | 2.08 | 0.224 |
| pN2 | 1.67 | 0.9 | 3.12 | 0.107 |

| **TLS-TD** | **OS** | | | |
|---|---|---|---|---|
| | HR | 95%CI lower | 95%CI upper | p-value |
| high | 1 | | | |
| low | 1.22 | 0.87 | 1.71 | 0.244 |
| pT1 | 1 | | | |
| pT2 | 1.23 | 0.85 | 1.8 | 0.272 |
| pT3 | 1.66 | 1.02 | 2.71 | 0.041 |
| pT4 | 3.05 | 1.58 | 5.92 | 0.0009 |
| pN0 | 1 | | | |
| pN1 | 1.06 | 0.76 | 1.48 | 0.403 |
| pN2 | 1.22 | 0.76 | 1.98 | 0.243 |

| **TLS-TD** | **PFS** | | | |
|---|---|---|---|---|
| | HR | 95%CI lower | 95%CI upper | p-value |
| high | 1 | | | |
| low | 1.92 | 1.17 | 3.14 | 0.009 |
| pT1 | 1 | | | |
| pT2 | 1.05 | 0.63 | 1.75 | 0.85 |
| pT3 | 2.44 | 1.34 | 4.41 | 0.003 |
| pT4 | 2.28 | 0.89 | 5.86 | 0.088 |
| pN0 | 1 | | | |
| pN1 | 1.94 | 1.28 | 2.93 | 0.002 |
| pN2 | 1.82 | 0.97 | 3.4 | 0.061 |

| **NECR-TD** | **CSS** | | | |
|---|---|---|---|---|
| | HR | 95%CI lower | 95%CI upper | p-value |
| high | 1 | | | |
| low | 1.72 | 1.13 | 2.62 | 0.012 |
| pT1 | 1 | | | |
| pT2 | 1.17 | 0.69 | 2 | 0.555 |
| pT3 | 2.03 | 1.05 | 3.91 | 0.035 |
| pT4 | 3.9 | 1.65 | 9.19 | 0.002 |
| pN0 | 1 | | | |
| pN1 | 1.22 | 0.78 | 1.91 | 0.372 |
| pN2 | 1.58 | 1.13 | 2.62 | 0.151 |

| **NECR-TD** | **OS** | | | |
|---|---|---|---|---|
| | HR | 95%CI lower | 95%CI upper | p-value |
| high | 1 | | | |
| low | 1.3 | 0.96 | 1.73 | 0.092 |
| pT1 | 1 | | | |
| pT2 | 1.22 | 0.84 | 1.79 | 0.286 |
| pT3 | 1.68 | 1.03 | 2.73 | 0.037 |
| pT4 | 3.04 | 1.57 | 5.91 | 0.001 |
| pN0 | 1 | | | |
| pN1 | 1.03 | 0.75 | 1.44 | 0.840 |
| pN2 | 1.29 | 0.73 | 1.91 | 0.494 |

| **NECR-TD** | **PFS** | | | |
|---|---|---|---|---|
| | HR | 95%CI lower | 95%CI upper | p-value |
| high | 1 | | | |
| low | 1.46 | 0.98 | 2.16 | 0.062 |
| pT1 | 1 | | | |
| pT2 | 1.07 | 0.64 | 1.78 | 0.802 |
| pT3 | 2.54 | 1.4 | 4.6 | 0.002 |
| pT4 | 2.6 | 1.02 | 6.67 | 0.046 |
| pN0 | 1 | | | |
| pN1 | 1.83 | 1.21 | 2.75 | 0.004 |
| pN2 | 1.77 | 0.95 | 3.31 | 0.072 |

| **T/NR** | CSS | | | |
|---|---|---|---|---|
| | HR | 95%CI lower | 95%CI upper | p-value |
| high | 1 | | | |
| low | 2.45 | 1.46 | 4.1 | 0.0007 |
| pT1 | 1 | | | |
| pT2 | 1.2 | 0.71 | 2.05 | 0.498 |
| pT3 | 1.9 | 0.98 | 3.67 | 0.056 |
| pT4 | 3.88 | 1.62 | 9.28 | 0.002 |
| pN0 | 1 | | | |
| pN1 | 1.32 | 0.84 | 2.07 | 0.223 |
| pN2 | 1.51 | 0.8 | 2.85 | 0.201 |

| **T/NR** | OS | | | |
|---|---|---|---|---|
| | HR | 95%CI lower | 95%CI upper | p-value |
| high | 1 | | | |
| low | 1.47 | 1.06 | 2.03 | 0.02 |
| pT1 | 1 | | | |
| pT2 | 1.24 | 0.85 | 1.81 | 0.257 |
| pT3 | 1.64 | 1 | 2.66 | 0.048 |
| pT4 | 3.24 | 1.67 | 6.32 | 0.0005 |
| pN0 | 1 | | | |
| pN1 | 1.06 | 0.77 | 1.48 | 0.713 |
| pN2 | 1.21 | 0.75 | 1.95 | 0.441 |

| **T/NR** | PFS | | | |
|---|---|---|---|---|
| | HR | 95%CI lower | 95%CI upper | p-value |
| high | 1 | | | |
| low | 1.9 | 1.2 | 3.01 | 0.006 |
| pT1 | 1 | | | |
| pT2 | 1.06 | 0.64 | 1.77 | 0.812 |
| pT3 | 2.36 | 1.3 | 4.28 | 0.005 |
| pT4 | 2.52 | 0.97 | 6.53 | 0.057 |
| pN0 | 1 | | | |
| pN1 | 1.9 | 1.26 | 2.87 | 0.002 |
| pN2 | 1.74 | 0.93 | 3.28 | 0.085 |

| **TLS-TD + NECR-TD** | **CSS** | | | |
|---|---|---|---|---|
| | HR | 95%CI lower | 95%CI upper | p-value |
| T high, N low | 1 | | | |
| T high + N high / T low + N low | 2 | 0.84 | 4.79 | 0.116 |
| T low + N high | 3.29 | 1.4 | 7.71 | 0.006 |
| pT1 | 1 | | | |
| pT2 | 1.14 | 0.67 | 1.94 | 0.63 |
| pT3 | 1.99 | 1.03 | 3.84 | 0.041 |
| pT4 | 3.46 | 1.47 | 8.18 | 0.005 |
| pN0 | 1 | | | |
| pN1 | 1.35 | 0.86 | 2.11 | 0.196 |
| pN2 | 1.61 | 0.87 | 3 | 0.131 |

| **TLS-TD + NECR-TD** | **OS** | | | |
|---|---|---|---|---|
| | HR | 95%CI lower | 95%CI upper | p-value |
| T high, N low | 1 | | | |
| T high + N high / T low + N low | 1.23 | 0.74 | 2.04 | 0.417 |
| T low + N high | 1.54 | 0.94 | 2.53 | 0.089 |
| pT1 | 1 | | | |
| pT2 | 1.22 | 0.83 | 1.77 | 0.31 |
| pT3 | 1.66 | 1.02 | 2.7 | 0.041 |
| pT4 | 3.01 | 1.55 | 5.83 | 0.001 |
| pN0 | 1 | | | |
| pN1 | 1.07 | 0.77 | 1.49 | 0.696 |
| pN2 | 1.21 | 0.75 | 1.95 | 0.430 |

| **TLS-TD + NECR-TD** | **PFS** | | | |
|---|---|---|---|---|
| | HR | 95%CI lower | 95%CI upper | p-value |
| T high, N low | 1 | | | |
| T high + N high / T low + N low | 1.87 | 0.83 | 4.19 | 0.129 |
| T low + N high | 2.87 | 1.3 | 6.35 | 0.009 |
| pT1 | 1 | | | |
| pT2 | 1.02 | 0.61 | 1.7 | 0.934 |
| pT3 | 2.42 | 1.33 | 4.41 | 0.004 |
| pT4 | 2.35 | 0.92 | 6.04 | 0.074 |
| pN0 | 1 | | | |
| pN1 | 1.97 | 1.3 | 2.97 | 0.001 |
| pN2 | 1.79 | 0.96 | 3.34 | 0.067 |

**Table S6 Results of univariate Cox regression analysis for clinical variables in lung adenocarcinoma cohort. Related to Figure 7**

| **AGE** | **CSS** | | | |
|---|---|---|---|---|
| | HR | 95%CI lower | 95%CI upper | p-value |
| each 10 years increase | 0.96 | 0.78 | 1.17 | 0.686 |

| **AGE** | **PFS** | | | |
|---|---|---|---|---|
| | HR | 95%CI lower | 95%CI upper | p-value |
| each 10 years increase | 1.09 | 0.91 | 1.29 | 0.344 |

| **AGE** | **OS** | | | |
|---|---|---|---|---|
| | HR | 95%CI lower | 95%CI upper | p-value |
| each 10 years increase | 1.08 | 0.92 | 1.26 | 0.353 |

| **SEX** | **CSS** | | | |
|---|---|---|---|---|
| | HR | 95%CI lower | 95%CI upper | p-value |
| female | 1 | | | |
| male | 0.97 | 0.64 | 1.47 | 0.883 |

| **SEX** | **PFS** | | | |
|---|---|---|---|---|
| | HR | 95%CI lower | 95%CI upper | p-value |
| female | 1 | | | |
| male | 1 | 0.7 | 1.46 | 0.96 |

| **SEX** | **OS** | | | |
|---|---|---|---|---|
| | HR | 95%CI lower | 95%CI upper | p-value |
| female | 1 | | | |
| male | 1.07 | 0.78 | 1.47 | 0.658 |

| **SMOKING HISTORY** | **CSS** | | | |
|---|---|---|---|---|
| | HR | 95%CI lower | 95%CI upper | p-value |
| no | 1 | | | |
| yes | 1.13 | 0.6 | 2.15 | 0.691 |

| **SMOKING HISTORY** | **PFS** | | | |
|---|---|---|---|---|
| | HR | 95%CI lower | 95%CI upper | p-value |
| no | 1 | | | |
| yes | 1 | 0.58 | 1.74 | 0.982 |

| **SMOKING HISTORY** | **OS** | | | |
|---|---|---|---|---|
| | HR | 95%CI lower | 95%CI upper | p-value |
| no | 1 | | | |
| yes | 0.93 | 0.59 | 1.48 | 0.754 |

**Table S7 Results of univariate Cox regression analysis for clinical variables in lung squamous cell carcinoma cohort. Related to Figure 7**

| **AGE** | **CSS** | | | |
|---|---|---|---|---|
| | HR | 95%CI lower | 95%CI upper | p-value |
| each 10 years increase | 1.04 | 0.84 | 1.28 | 0.719 |

| **AGE** | **PFS** | | | |
|---|---|---|---|---|
| | HR | 95%CI lower | 95%CI upper | p-value |
| each 10 years increase | 0.98 | 0.81 | 1.19 | 0.829 |

| **AGE** | **OS** | | | |
|---|---|---|---|---|
| | HR | 95%CI lower | 95%CI upper | p-value |
| each 10 years increase | 1.14 | 0.97 | 1.33 | 0.108 |

| **SEX** | **CSS** | | | |
|---|---|---|---|---|
| | HR | 95%CI lower | 95%CI upper | p-value |
| female | 1 | | | |
| male | 1.3 | 0.82 | 2.07 | 0.266 |

| **SEX** | **PFS** | | | |
|---|---|---|---|---|
| | HR | 95%CI lower | 95%CI upper | p-value |
| female | 1 | | | |
| male | 1.42 | 0.9 | 2.23 | 0.129 |

| **SEX** | **OS** | | | |
|---|---|---|---|---|
| | HR | 95%CI lower | 95%CI upper | p-value |
| female | 1 | | | |
| male | 1.16 | 0.83 | 1.62 | 0.379 |

| **SMOKING HISTORY** | **CSS** | | | |
|---|---|---|---|---|
| | HR | 95%CI lower | 95%CI upper | p-value |
| no | 1 | | | |
| yes | 0.65 | 0.21 | 2.06 | 0.466 |

| **SMOKING HISTORY** | **PFS** | | | |
|---|---|---|---|---|
| | HR | 95%CI lower | 95%CI upper | p-value |
| no | 1 | | | |
| yes | 0.5 | 0.2 | 1.23 | 0.131 |

| **SMOKING HISTORY** | **OS** | | | |
|---|---|---|---|---|
| | HR | 95%CI lower | 95%CI upper | p-value |
| no | 1 | | | |
| yes | 0.65 | 0.29 | 1.47 | 0.304 |

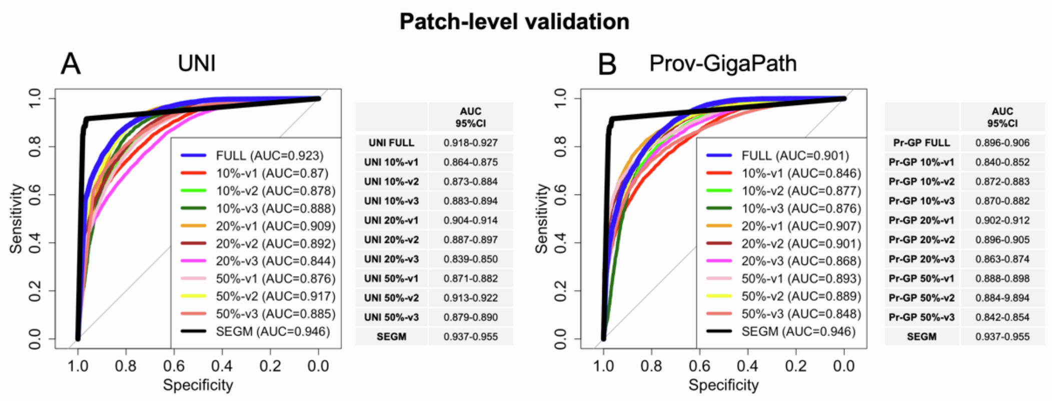


| | AUC 95%CI |
|---|---|
| UNI FULL | 0.918-0.927 |
| UNI 10%-v1 | 0.864-0.875 |
| UNI 10%-v2 | 0.873-0.884 |
| UNI 10%-v3 | 0.883-0.894 |
| UNI 20%-v1 | 0.904-0.914 |
| UNI 20%-v2 | 0.887-0.897 |
| UNI 20%-v3 | 0.839-0.850 |
| UNI 50%-v1 | 0.871-0.882 |
| UNI 50%-v2 | 0.913-0.922 |
| UNI 50%-v3 | 0.879-0.890 |
| SEGM | 0.937-0.955 |

| | AUC 95%CI |
|---|---|
| Pr-GP FULL | 0.896-0.906 |
| Pr-GP 10%-v1 | 0.840-0.852 |
| Pr-GP 10%-v2 | 0.872-0.883 |
| Pr-GP 10%-v3 | 0.870-0.882 |
| Pr-GP 20%-v1 | 0.902-0.912 |
| Pr-GP 20%-v2 | 0.896-0.905 |
| Pr-GP 20%-v3 | 0.863-0.874 |
| Pr-GP 50%-v1 | 0.888-0.898 |
| Pr-GP 50%-v2 | 0.884-9.894 |
| Pr-GP 50%-v3 | 0.842-0.854 |
| SEGM | 0.937-0.955 |

**Figure S1 Lung cancer subtyping task: test of the supervised classification models (Related to Figures 4 and 5)** based on A) UNI foundational feature extractor, B) Prov-GigaPath (Pr-GP) foundational feature extractor; compared to the fully supervised model developed in this study (SEGM). Independent test dataset: UKK L1 SEGM. Principle: Patch-level, AUROC analysis. For both model, models trained on full training data (FULL) and on limited training data (e.g., 10%, 20%, etc.) are shown.

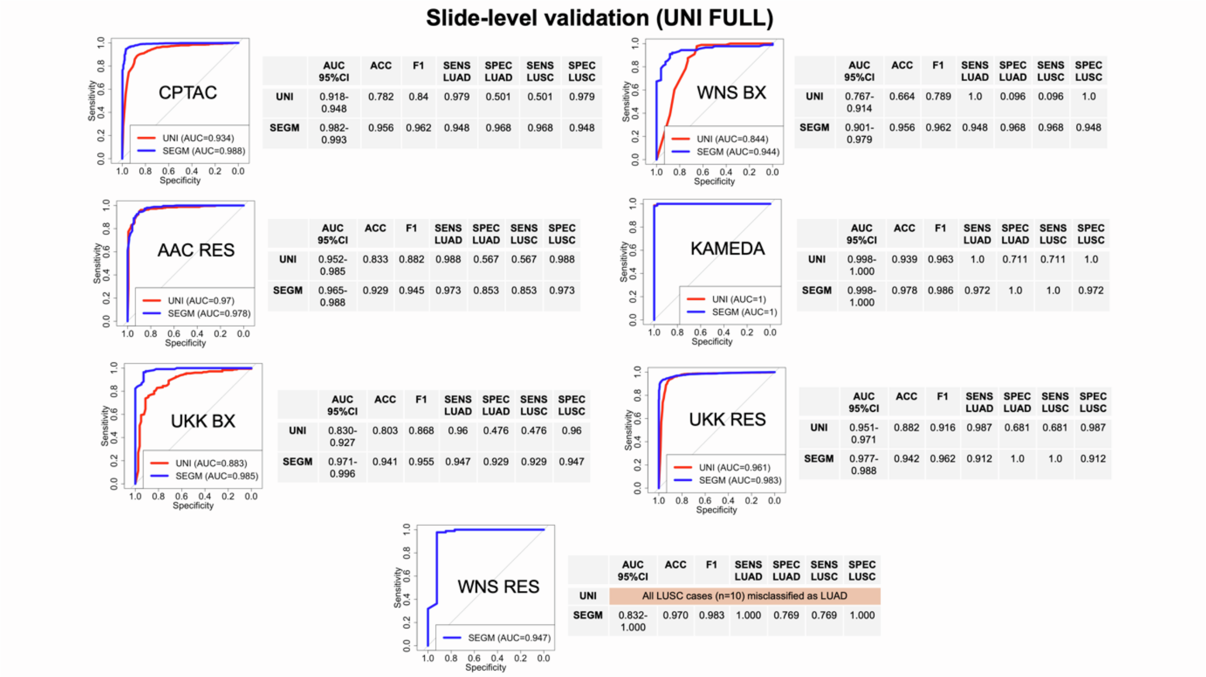


CPTAC

| | AUC 95%CI | ACC | F1 | SENS LUAD | SPEC LUAD | SENS LUSC | SPEC LUSC |
|---|---|---|---|---|---|---|---|
| UNI | 0.918-0.948 | 0.782 | 0.84 | 0.979 | 0.501 | 0.501 | 0.979 |
| SEGM | 0.982-0.993 | 0.956 | 0.962 | 0.948 | 0.968 | 0.968 | 0.948 |

WNS BX

| | AUC 95%CI | ACC | F1 | SENS LUAD | SPEC LUAD | SENS LUSC | SPEC LUSC |
|---|---|---|---|---|---|---|---|
| UNI | 0.767-0.914 | 0.664 | 0.789 | 1.0 | 0.096 | 0.096 | 1.0 |
| SEGM | 0.901-0.979 | 0.956 | 0.962 | 0.948 | 0.968 | 0.968 | 0.948 |

AAC RES

| | AUC 95%CI | ACC | F1 | SENS LUAD | SPEC LUAD | SENS LUSC | SPEC LUSC |
|---|---|---|---|---|---|---|---|
| UNI | 0.952-0.985 | 0.833 | 0.882 | 0.988 | 0.567 | 0.567 | 0.988 |
| SEGM | 0.965-0.988 | 0.929 | 0.945 | 0.973 | 0.853 | 0.853 | 0.973 |

KAMEDA

| | AUC 95%CI | ACC | F1 | SENS LUAD | SPEC LUAD | SENS LUSC | SPEC LUSC |
|---|---|---|---|---|---|---|---|
| UNI | 0.998-1.000 | 0.939 | 0.963 | 1.0 | 0.711 | 0.711 | 1.0 |
| SEGM | 0.998-1.000 | 0.978 | 0.986 | 0.972 | 1.0 | 1.0 | 0.972 |

UKK BX

| | AUC 95%CI | ACC | F1 | SENS LUAD | SPEC LUAD | SENS LUSC | SPEC LUSC |
|---|---|---|---|---|---|---|---|
| UNI | 0.830-0.927 | 0.803 | 0.868 | 0.96 | 0.476 | 0.476 | 0.96 |
| SEGM | 0.971-0.996 | 0.941 | 0.955 | 0.947 | 0.929 | 0.929 | 0.947 |

UKK RES

| | AUC 95%CI | ACC | F1 | SENS LUAD | SPEC LUAD | SENS LUSC | SPEC LUSC |
|---|---|---|---|---|---|---|---|
| UNI | 0.951-0.971 | 0.882 | 0.916 | 0.987 | 0.681 | 0.681 | 0.987 |
| SEGM | 0.977-0.988 | 0.942 | 0.962 | 0.912 | 1.0 | 1.0 | 0.912 |

WNS RES

| | AUC 95%CI | ACC | F1 | SENS LUAD | SPEC LUAD | SENS LUSC | SPEC LUSC |
|---|---|---|---|---|---|---|---|
| UNI | All LUSC cases (n=10) misclassified as LUAD | | | | | | |
| SEGM | 0.832-1.000 | 0.970 | 0.983 | 1.000 | 0.769 | 0.769 | 1.000 |

**Figure S2 Lung cancer subtyping task: extended test of a supervised classification model based on UNI foundational feature extractor** (trained on full training data; see Fig. S1), **Related to Figures 4 and 5**; compared to the fully supervised model developed in this study (SEGM). Independent test dataset: slide-level test datasets (CPTAC, AAC RES, WNS RES, UKK RES, WNS BX, UKK BX). Principle: slide-level, AUROC analysis, further accuracy metrics (ACC – overall accuracy, F1 – F1 score, SENS – Sensitivity, SPEC - specificity). Substantial accuracy problems can be seen for biopsy cohorts. Abbreviations: LUAD – lung adenocarcinoma, LUSC – lung squamous cell carcinoma.

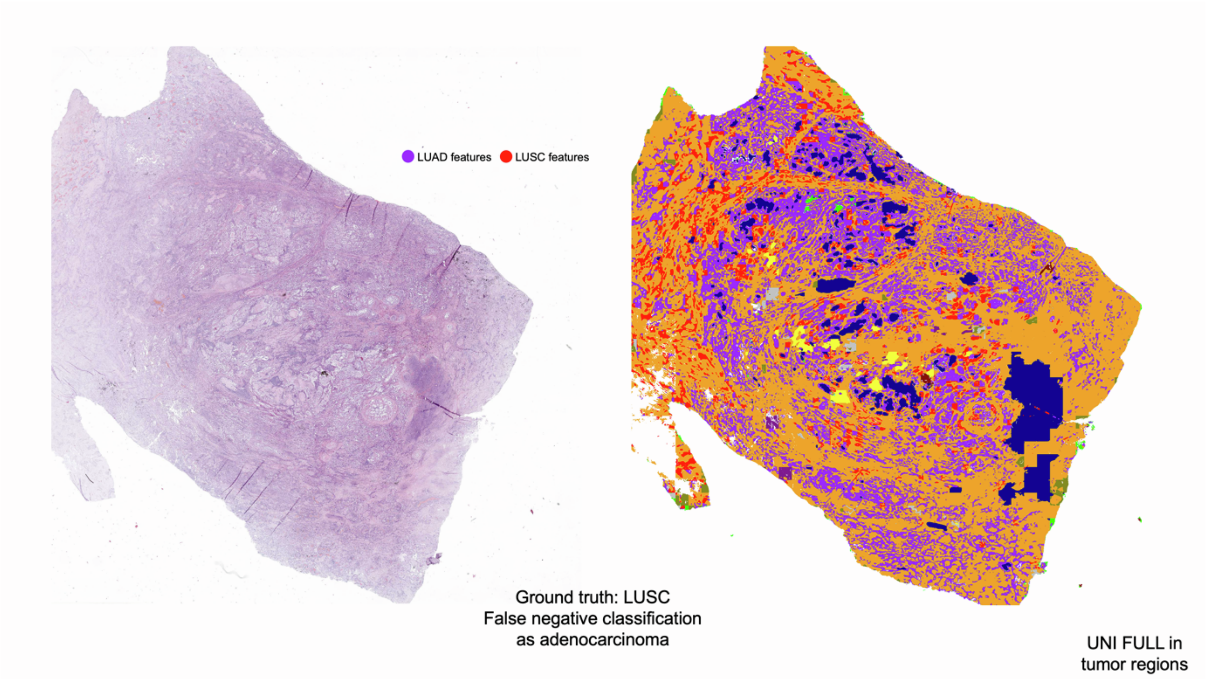


**Figure S3 Example of the whole slide image inference using UNI FULL supervised classification model from Fig. S1 and S2. Related to Figures 4 and 5** The case is a lung squamous cell carcinoma (LUSC) misclassified as lung adenocarcinoma (LUAD). Common pattern of misclassification is depicted: small LUSC structures are being recognized as LUAD.

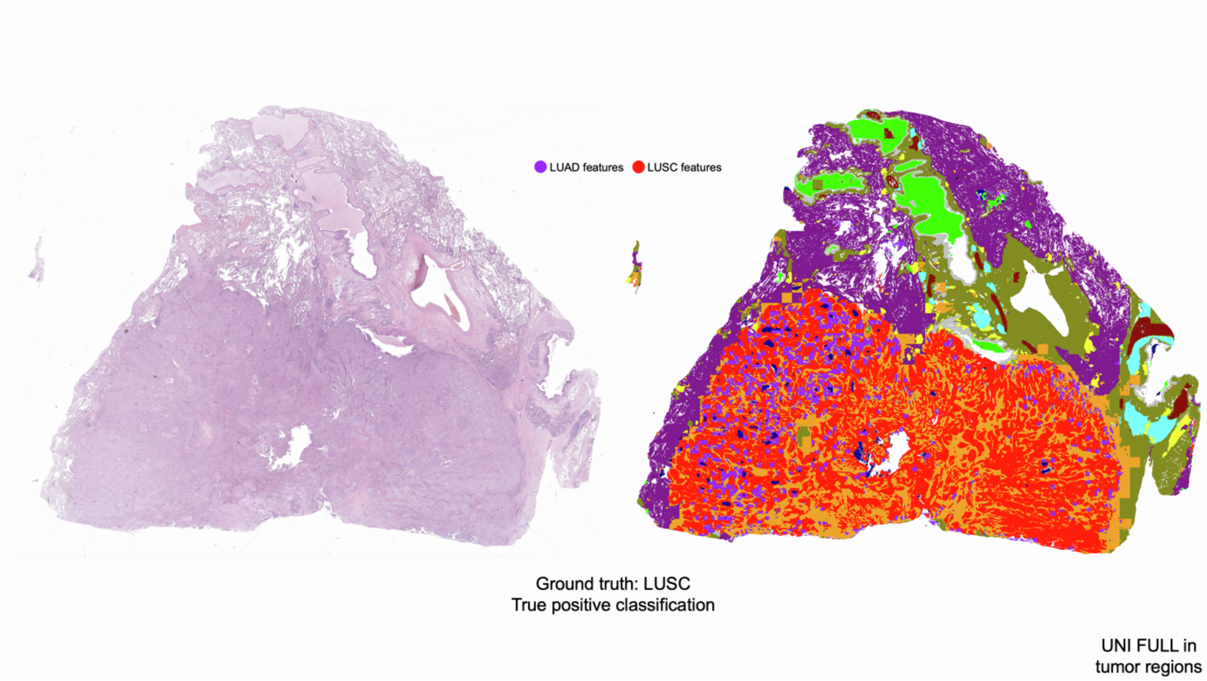


**Figure S4 Example of the whole slide image inference using UNI FULL supervised classification model from Fig. S1 and S2. Related to Figures 4 and 5**The case is a lung squamous cell carcinoma (LUSC) misclassified as lung adenocarcinoma (LUAD). Common pattern of misclassification is depicted: a clear LUSC morphology is classified as LUAD due to very restricted context size of the patch. The foundation models available today only allow analysis in patches with a size of 224x224 px. For comparison, the fully supervised segmentation model developed in this study utilizes patch size 512 px which allows for much more morphological context.

### Slide-level validation (CLAM/UNI)

**CPTAC**

| | AUC | AUC 95%CI | AUC Mean 10 folds | AUC Range 10 fold |
|---|---|---|---|---|
| UNI CLAM | - | - | 0.956 | 0.936-0.967 |
| SEGM | 0.988 | 0.982-0.993 | - | - |

**WNS BX**

| | AUC | AUC 95%CI | AUC Mean 10 folds | AUC Range 10 fold |
|---|---|---|---|---|
| UNI CLAM | - | - | 0.886 | 0.835-0.925 |
| SEGM | 0.944 | 0.901-0.979 | - | - |

**AAC RES**

| | AUC | AUC 95%CI | AUC Mean 10 folds | AUC Range 10 fold |
|---|---|---|---|---|
| UNI CLAM | - | - | 0.985 | 0.980-0.988 |
| SEGM | 0.978 | 0.965-0.988 | - | - |

**KAMEDA**

| | AUC | AUC 95%CI | AUC Mean 10 folds | AUC Range 10 fold |
|---|---|---|---|---|
| UNI CLAM | - | - | 0.999 | 0.998-1.0 |
| SEGM | 1.0 | 0.998-1.0 | - | - |

**UKK BX**

| | AUC | AUC 95%CI | AUC Mean 10 folds | AUC Range 10 fold |
|---|---|---|---|---|
| UNI CLAM | - | - | 0.918 | 0.859-0.954 |
| SEGM | 0.985 | 0.971-0.996 | - | - |

**UKK RES**

| | AUC | AUC 95%CI | AUC Mean 10 folds | AUC Range 10 fold |
|---|---|---|---|---|
| UNI CLAM | - | - | 0.986 | 0.983-0.989 |
| SEGM | 0.983 | 0.977-0.988 | - | - |

**WNS RES**

| | AUC | AUC 95%CI | AUC Mean 10 folds | AUC Range 10 fold |
|---|---|---|---|---|
| UNI CLAM | - | - | 0.927 | 0.870-0.983 |
| SEGM | 0.947 | 0.832-1.0 | - | - |

**Figure S5 Lung cancer subtyping task: extended test of a CLAM model trained using UNI foundational feature extractor** (trained using full training data), **Related to Figures 4 and 5**; compared to the fully supervised model developed in this study (SEGM). Independent test dataset: slide-level test datasets (CPTAC, AAC RES, WNS RES, UKK RES, WNS BX, UKK BX). Principle: slide-level, AUROC analysis, CLAM models were trained using original 10-fold principle, the AUROC range for all 10 folds is provided. Substantial accuracy problems can be seen for biopsy cohorts. Abbreviations: LUAD – lung adenocarcinoma, LUSC – lung squamous cell carcinoma.

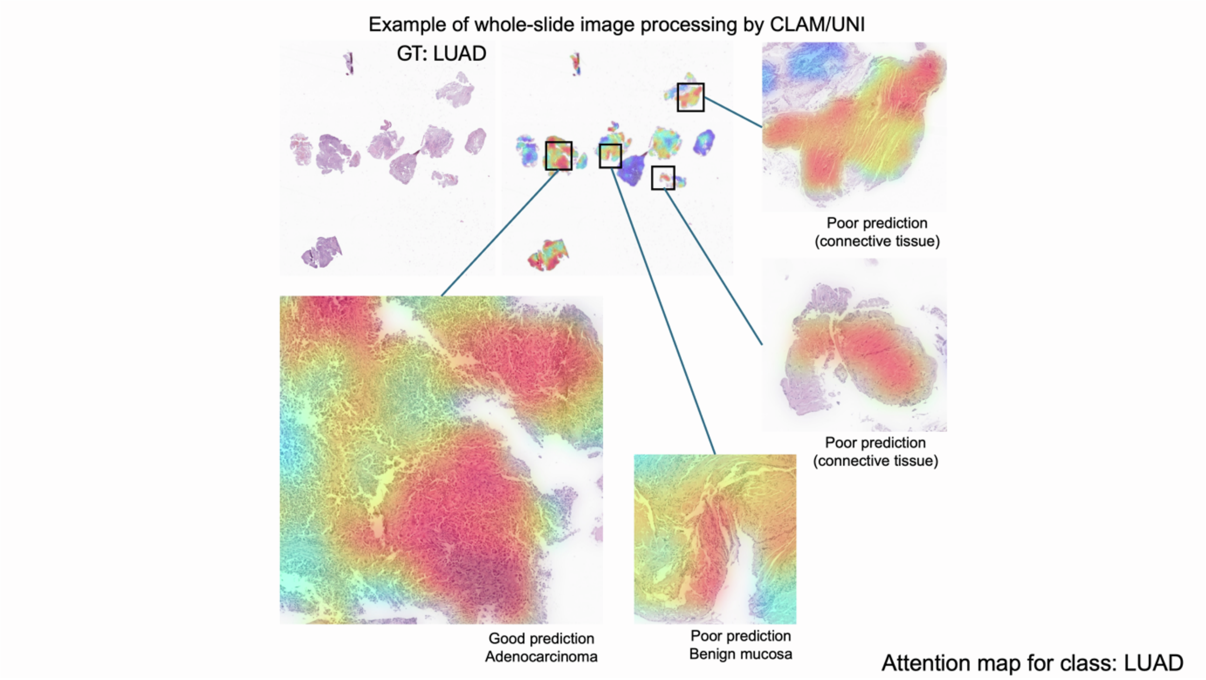


**Figure S6 Detailed morphological analysis of subtype predictions by CLAM/UNI model in a biopsy case with lung adenocarcinoma (LUAD). Related to Figures 4 and 5.** The attention map which are used for the final subtype prediction is shown for LUAD class. Many regions with high attention levels of being LUAD are poor predictions (benign tissue, e.g. connective tissue, cartilage, benign mucosa, metaplasia, etc.). The same patterns can be seen in resection cases. Abbreviations: GT – ground truth.

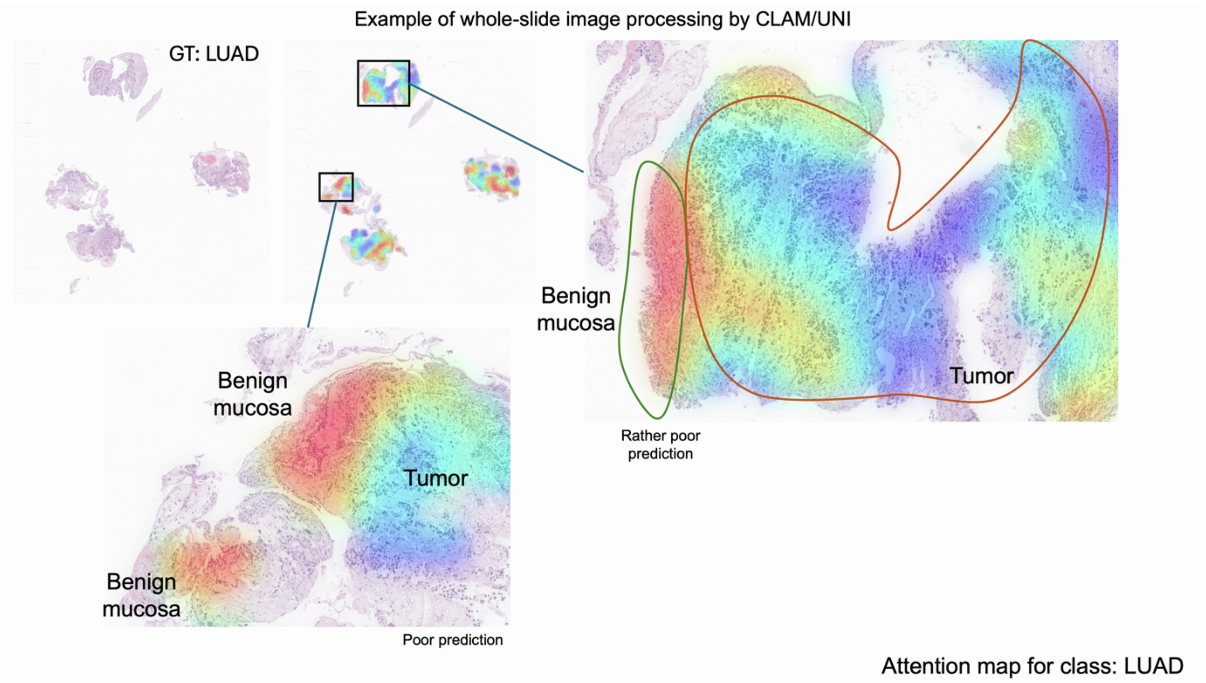


**Figure S7 Detailed morphological analysis of subtype predictions by CLAM/UNI model in a biopsy case with lung adenocarcinoma (LUAD). Related to Figures 4 and 5.** The attention map which are used for the final subtype prediction is shown for LUAD class. Many regions with high attention levels of being LUAD are poor predictions (benign tissue, e.g. connective tissue, cartilage, benign mucosa, metaplasia, etc.). The same patterns can be seen in resection cases. Abbreviations: GT – ground truth.

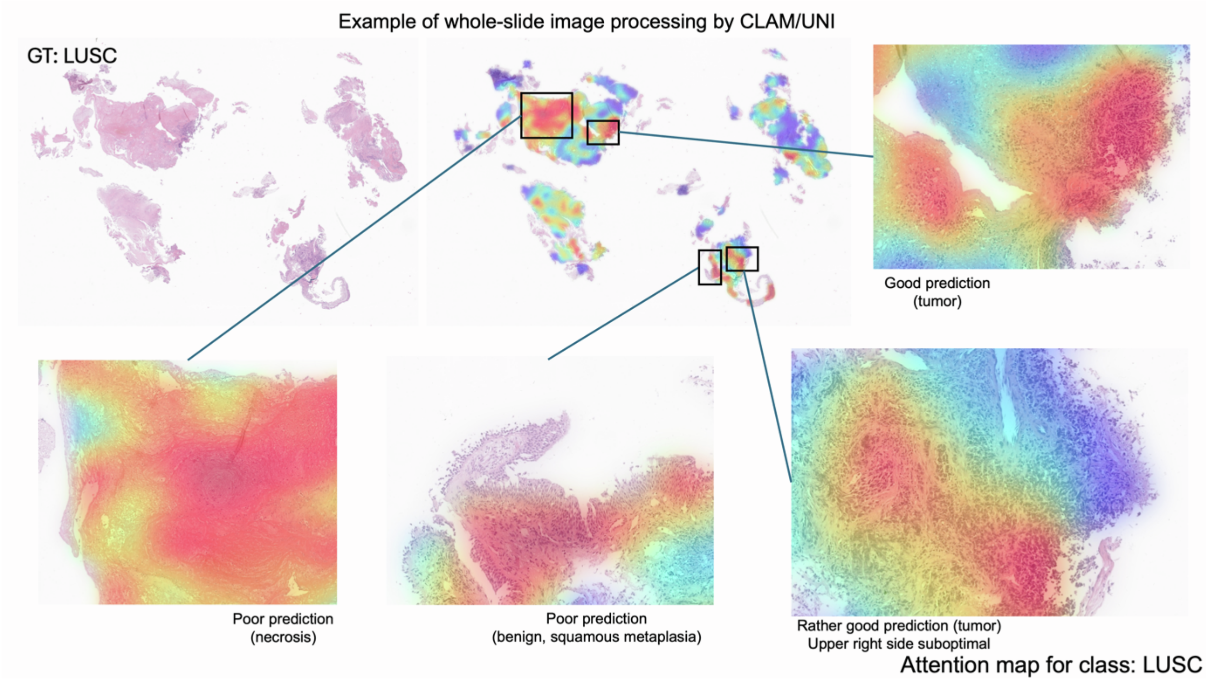


**Figure S8 Detailed morphological analysis of subtype predictions by CLAM/UNI model in a biopsy case with lung squamous cell carcinoma (LUSC). Related to Figures 4 and 5.** The attention map which are used for the final subtype prediction is shown for LUSC class. Many regions with high attention levels of being LUSC are poor predictions (benign tissue, e.g. connective tissue, cartilage, benign mucosa, metaplasia, etc.). The same patterns can be seen in resection cases. Abbreviations: GT – ground truth.

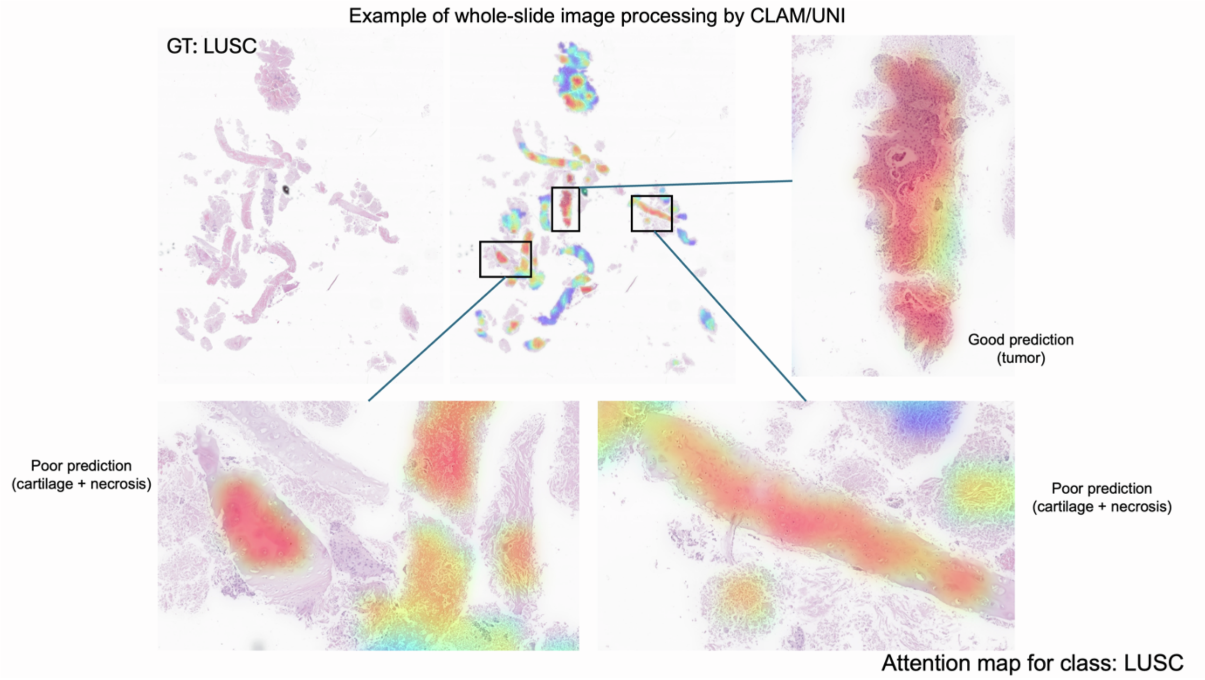


**Figure S9 Detailed morphological analysis of subtype predictions by CLAM/UNI model in a biopsy case with lung squamous cell carcinoma (LUSC). Related to Figures 4 and 5.** The attention map which are used for the final subtype prediction is shown for LUSC class. Many regions with high attention levels of being LUSC are poor predictions (benign tissue, e.g. connective tissue, cartilage, benign mucosa, metaplasia, etc.). The same patterns can be seen in resection cases. Abbreviations: GT – ground truth.

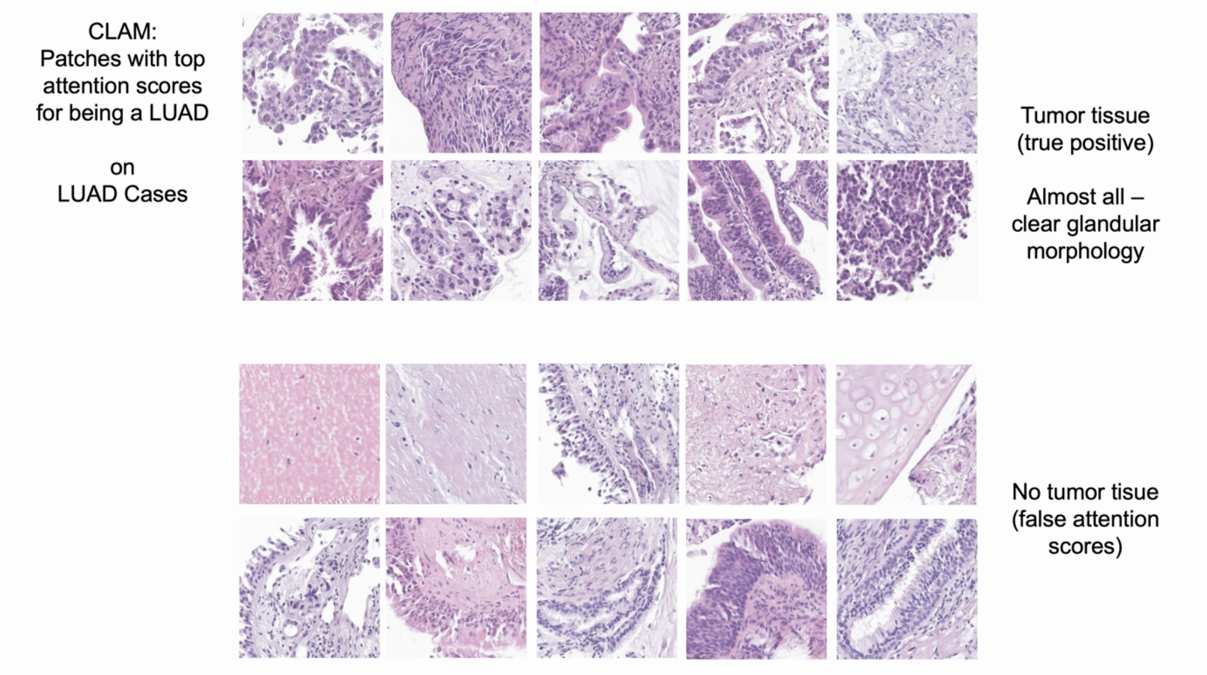


**Figure S10 Top image patches based on attention of the trained CLAM model used for final classification. Related to Figures 4 and 5.** Patches with top attention scores shown for subtype: LUAD. Ground truth of cases: LUAD. Multiple cases are summarized. Among the top attention regions true positive for presence of tumor tissue (upper two rows), mostly LUAD regions with glandular differentiation (=clear LUAD morphology) are present and detected by CLAM. Importantly, multiple regions over many whole-slide images with top attention scores for being a LUAD do not contain any tumor tissue (lower two rows) and therefore are false features/biases learnt by the CLAM model (see Discussion). This corresponds to analysis presented in Figures S6-S9.

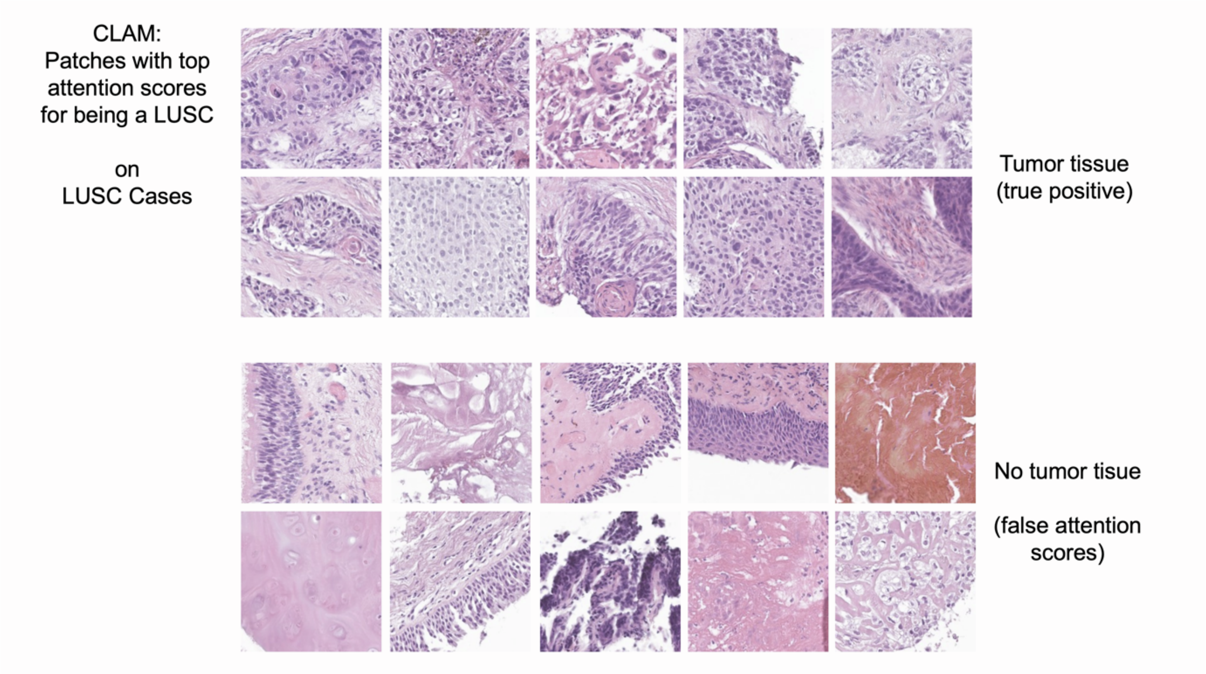


**Figure S11 Top image patches based on attention of the trained CLAM model used for final classification. Related to Figures 4 and 5.** Patches with top attention scores shown for subtype: LUSC. Ground truth of cases: LUSC. Multiple cases are summarized. Among the top attention regions true positive for presence of tumor tissue (upper two rows), cornification is often present (very specific but not sensitive morphological feature of LUSC). Importantly, multiple regions over many whole-slide images with top attention scores for being a LUSC do not contain any tumor tissue (lower two rows) and therefore are false features/biases learnt by the CLAM model (see Discussion). This corresponds to analysis presented in Figures S6-S9.

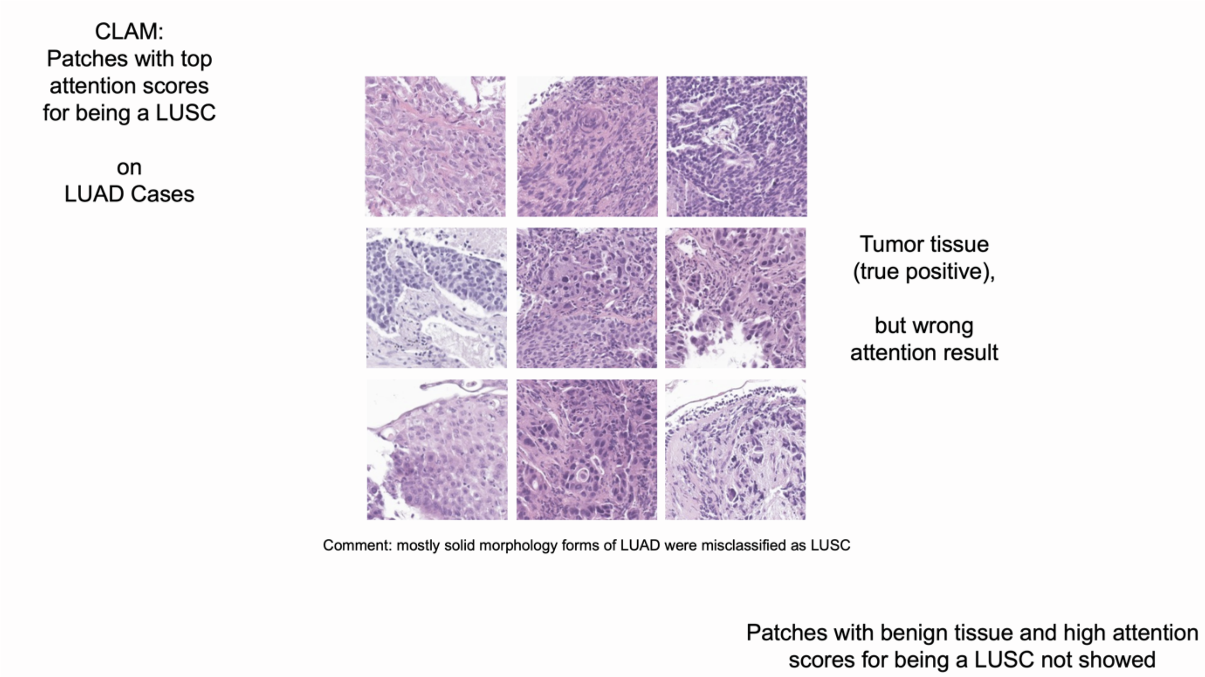


**Figure S12 Top image patches based on attention of the trained CLAM model used for final classification. Related to Figures 4 and 5.** Patches with top attention scores shown for subtype: LUSC. Ground truth of cases: LUAD. These are the regions within LUAD cases that were misclassified by CLAM model as LUSC. The images represent typical morphology with solid areas without glandular differentiation, a common feature of cases referred as "challenging" in Figure 5. This emphasizes that independent of algorithm (fully supervised pixel-wise or CLAM-based), this category of cases cannot be reliably classified based on solely image analysis of H&E-stained slides and requires additional immunohistochemical stains.

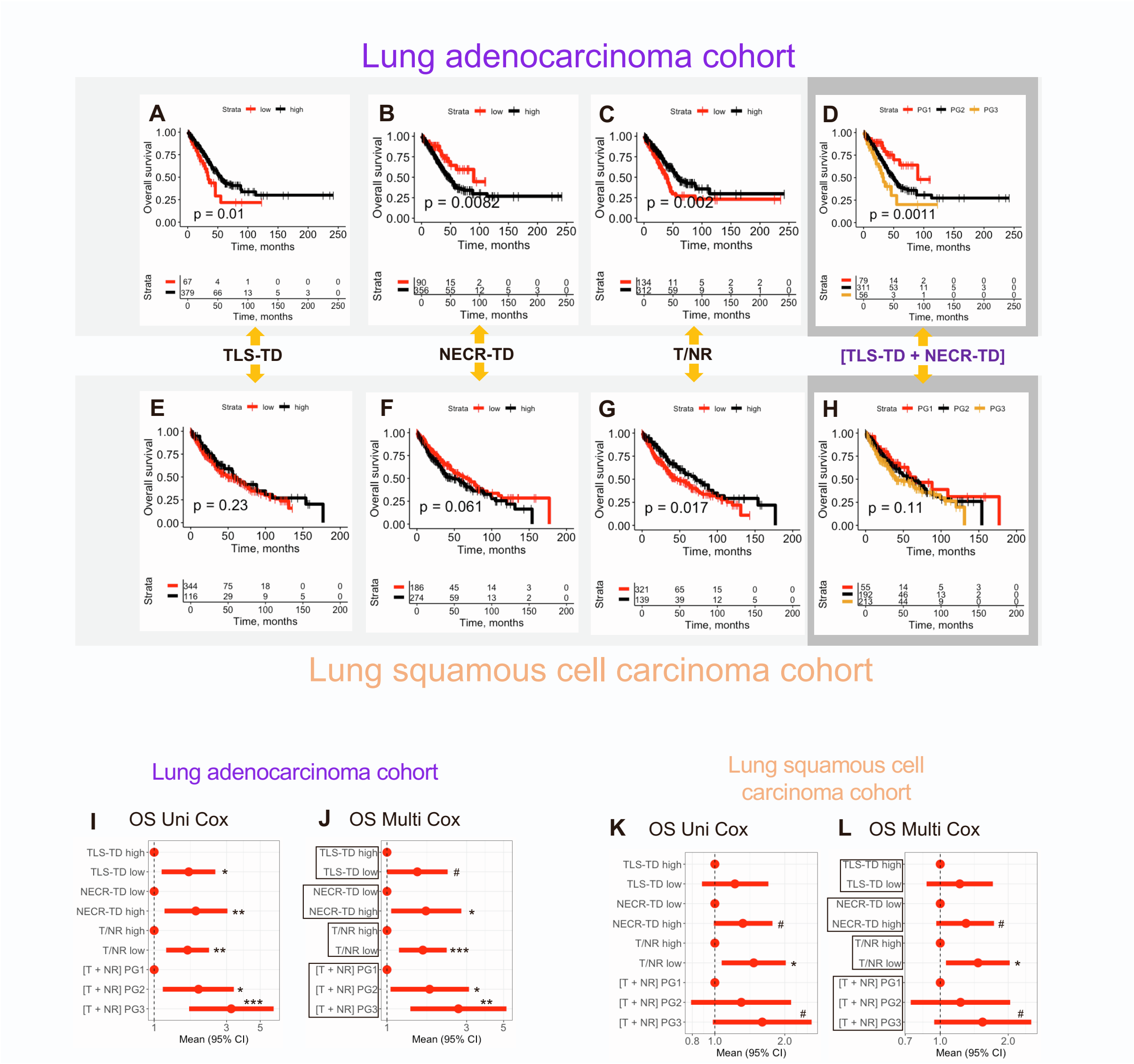


**Figure S13 Evaluation of prognostic role of new AI-based prognostic parameters for overall survival (OS) endpoint**. **Related to Figures 6 and 7 A-D.** Lung adenocarcinoma cohort: A – TLS-TD, B – NECR-TD, C – T/NR, D – [T+NR]. **E-H.** Lung squamous cell carcinoma cohort: E – TLS-TD, F – NECR-TD, G – T/NR, H – [T+NR]. The parameters TLS-TD, NECR-TD and T/NR are dichotomized using identified optimal cut-off to derive prognostic subgroups. **I.-L.** Results of univariate and multivariate Cox proportional hazard model analysis for new prognostic parameters. **I-J**: Lung adenocarcinoma cohort. **K-L**: Lung squamous cell carcinoma cohort. Comment: All multivariate models always include pT and pN-classification of the tumor and one prognostic parameter; therefore, one plot shows several multivariate models, one for each of prognostic parameter for easiness of visualization. The analyzed parameter is included in frame. Plots show Hazard Ratios (HR) and 95% confidence interval (95%CI). Abbreviations: # - p-level 0.05-0.1 (statistical trend), * p-level 0.01-0.05, ** p-level 0.001-0.01, *** p-level < 0.001. Detailed information to Uni- and Multivariate Cox analysis is provided in Tables A2-A5.
